# Is there a place for sigmoidoscopy in colorectal cancer screening? A systematic review and critical appraisal of cost-effectiveness models

**DOI:** 10.1371/journal.pone.0290353

**Published:** 2023-08-18

**Authors:** Leonie Diedrich, Melanie Brinkmann, Maren Dreier, Siegbert Rossol, Wendelin Schramm, Christian Krauth

**Affiliations:** 1 Institute for Epidemiology, Social Medicine and Health Systems Research, Hannover Medical School, Hannover, Germany; 2 Department of Internal Medicine, Krankenhaus Nordwest, Frankfurt/M, Germany; 3 GECKO Institute for Medicine, Informatics and Economics, Heilbronn University, Heilbronn, Germany; Tehran University of Medical Sciences, ISLAMIC REPUBLIC OF IRAN

## Abstract

**Introduction:**

Screening for colorectal cancer (CRC) is effective in reducing both incidence and mortality. Colonoscopy and stool tests are most frequently used for this purpose. Sigmoidoscopy is an alternative screening measure with a strong evidence base. Due to its distinct characteristics, it might be preferred by subgroups. The aim of this systematic review is to analyze the cost-effectiveness of sigmoidoscopy for CRC screening compared to other screening methods and to identify influencing parameters.

**Methods:**

A systematic literature search for the time frame 01/2010–01/2023 was conducted using the databases MEDLINE, Embase, EconLit, Web of Science, NHS EED, as well as the Cost-Effectiveness Registry. Full economic analyses examining sigmoidoscopy as a screening measure for the general population at average risk for CRC were included. Incremental cost-effectiveness ratios were calculated. All included studies were critically assessed based on a questionnaire for modelling studies.

**Results:**

Twenty-five studies are included in the review. Compared to no screening, sigmoidoscopy is a cost-effective screening strategy for CRC. When modelled as a single measure strategy, sigmoidoscopy is mostly dominated by colonoscopy or modern stool tests. When combined with annual stool testing, sigmoidoscopy in 5-year intervals is more effective and less costly than the respective strategies alone. The results of the studies are influenced by varying assumptions on adherence, costs, and test characteristics.

**Conclusion:**

The combination of sigmoidoscopy and stool testing represents a cost-effective screening strategy that has not received much attention in current guidelines. Further research is needed that goes beyond a narrow focus on screening technology and models different, preference-based participation behavior in subgroups.

## Introduction

Colorectal cancer (CRC) is one of the most common tumorous diseases in developed countries: in 2020, it accounted for 10.3% of cancer cases and 9.9% of cancer-associated deaths in Europe, ranking third and second respectively [1]. Screening has the potential to reduce both CRC incidence and CRC mortality. Since most intestinal tumors develop along the adenoma-carcinoma sequence over a long period of time, CRC can be detected at early stages or even be prevented by removing precursor lesions [2]. The majority of European countries have implemented organized or opportunistic screening following the recommendation of the European Council [3]. The most common screening methods in Europe include immunochemical or guaiac-based fecal occult blood tests (FIT / gFOBT), as well as structural examinations of the bowel by colonoscopy. Newer methods, such as DNA testing or CT-colonography, are not regularly used in Europe [4, 5]. The European Colorectal Cancer Screening Guidelines Working Group recommends that individuals aged 50 to 74 years undergo annual to biennial FIT or gFOBT, which is low-cost and approved as a screening measure throughout the entire European Union [6, 7]. A meta-analysis of randomized controlled trials (RCTs) shows a significant reduction in CRC mortality with stool tests [8]. With their low sensitivity for adenomas, stool tests are primarily detection tests for cancer. A positive finding must be followed by a complete colonoscopy [9].

Endoscopic procedures, such as colonoscopy or sigmoidoscopy, are superior to non-invasive stool tests in terms of detecting CRC and adenomas [9]. Colonoscopy is considered the gold standard, as the procedure can be both diagnostic and therapeutic. During colonoscopy, which consists of an endoscopic visualization of the entire colon, precursor lesions can be directly removed. Sigmoidoscopy is an alternative endoscopic procedure during which only the distal segment of the bowel is examined. Although colonoscopy is part of CRC screening in several European countries, such as Germany or Poland, incidence and mortality reductions as a result of colonoscopy have so far been demonstrated only by observational studies [5, 10]. First results of an RCT examining colonoscopy in Poland, the Netherlands, and Sweden published in October 2022 indicated that, over a 10-year period, colonoscopy resulted in a smaller reduction in incidence compared with results from observational studies [11]. A reduction in mortality was not clearly demonstrated. However, it is possible that the effectiveness of colonoscopy could not be adequately surveyed because of the relatively short follow-up period and low participation rate (42%). Per-protocol analysis showed a greater incidence and mortality reduction [11]. For sigmoidoscopy, recent RCTs are available, which in a meta-analysis result in an incidence and mortality reduction of 24% and 26%, respectively [12].

Over the past few decades, with the growing implementation of colonoscopy in health care, sigmoidoscopy has increasingly fallen into disuse. In Europe, sigmoidoscopy is used for population-based screening only in the Italian region Piedmont [3], although it is stated in several European guidelines as a reasonable measure [4]. A possible reason for the shift away from sigmoidoscopy is its weakness in detecting proximal lesions compared to colonoscopy. However, due to a lack of evidence demonstrating the relevance of proximal lesions for CRC screening, it is questionable whether colonoscopy can be considered superior to sigmoidoscopy as a result of its ability to detect such neoplasms. When differentiated by location, colonoscopy is associated with no significant incidence and mortality reduction of proximal neoplasms [10, 13–15] or a significantly smaller reduction compared with distal carcinomas [16]. The reason for this may be the histological nature of the upper intestinal segment. This area more frequently sees the growth of sessile serrated adenomas, which are more difficult to detect and at the same time associated with a more rapid carcinogenesis. As a consequence, CRC is mostly detected at a higher stage in this bowel section. Only a minority of carcinomas arise from this form of precursor lesions along the alternated serrated pathway [17].

As all screening strategies incorporate specific advantages and disadvantages, there is no universally ideal method. The appropriateness of a screening strategy depends on many factors. For example, the setting, the available resources, and the willingness of the respective target population to participate must be taken into account [18]. In particular, participation rates are still low in Europe and often below the minimum target of 45% as defined by the European Union [6, 19].

Because the choice of screening modality is highly preference-sensitive, procedural characteristics are a critical determinant of anticipated participation. Reservations seem to be more prevalent with invasive procedures than with stool tests [20]. In the case of colonoscopy, patients often describe the extensive preparation (drinking up to 2 liters or more of laxatives and concurrent dietary restrictions) as burdensome. Furthermore, the necessary sedation leads to an inability to work on the day of the examination, and the need for an accompanying person creates additional organizational effort [21]. For sigmoidoscopy, on the other hand, an enema is usually sufficient as preparation and sedation is not strictly necessary [22]. Risks such as major bleeding or perforations are less pronounced compared to colonoscopy [23]. Due to its inherent characteristics, sigmoidoscopy may be better accepted by patients and is thus more likely to be utilized.

In the face of ever-evolving medicine, scarce resources, and low participation, the question emerges to which extent it may be appropriate to shift focus to sigmoidoscopy, an evidence-based strategy which is possibly preferred by subgroups. Decision-analytical modeling provides valuable information on optimal resource allocation, especially when the events of interest are far in the future. When costs and effects are contrasted, all current CRC screening methods appear to be cost-effective or even cost-saving compared to no screening offer. There is disagreement about which strategy is optimal in terms of cost-effectiveness [24–28]. Previous systematic reviews have focused on the question of the most cost-effective single strategy, mostly assuming perfect adherence. Since the potential of sigmoidoscopy lies in complementing less-sensitive procedures (stool test) or strategies with preference-related low participation (colonoscopy), such a consideration is insufficient. It is therefore useful to consider the performance of sigmoidoscopy in combination with other strategies, as well as to identify the most influential modeling parameters. The validity of the results depends on the representation of the natural history of the disease, the modeled effectiveness of screening, and the data used [29]. There are significant underlying structural and qualitative differences between models. However, this was only captured narratively [18] or pertained primarily to reporting quality [26, 28].

The aim of this systematic review is to analyze the cost-effectiveness of sigmoidoscopy for CRC screening either alone or in combination with other strategies compared to current and newer screening methods and to identify relevant influencing parameters. It is also the first review in the field of CRC screening to provide a comprehensive quality assessment of modeling studies using instruments by the International Society for Pharmacoeconomics and Outcomes Research (ISPOR) [30].

## Methods

The protocol for this systematic review was registered in Prospero (CRD42020189934). The methodology was based on the Preferred Reporting Items for Systematic Reviews and Meta-Analyses (PRISMA), though not all items are applicable to economic evaluations [31]. The research question was worded as follows:

*Is sigmoidoscopy in colorectal cancer screening cost-effective compared to other commonly used screening strategies and what are relevant factors influencing the results?*

### Search strategy

The databases MEDLINE, Embase, EconLit, Web of Science, the British National Health Service Economic Evaluation Database (NHS EED), as well as the Cost-Effectiveness Registry (Tufts Medical Center) were systematically searched in March 2020. The search was updated in August 2021 and January 2023, ultimately covering the time frame of 01/2010 to 01/2023. The search strategy consisted of 2 blocks, describing Colorectal Cancer Screening and Economic Evaluation respectively (S1 Fig). In each block, various search terms, including both controlled vocabulary (MeSH Terms) and free terms, were linked with the Boolean operator “OR”. The blocks were then combined with the operator “AND”. The search syntax for each database can be found in the appendix (S2 Table).

### Study selection

Studies were considered if they met the a priori defined inclusion criteria. Only full economic analyses that examined the general population at average risk for CRC and compared sigmoidoscopy as a screening strategy to either no screening or at least one other generally available screening measure were eligible for inclusion. Studies were included if they reported long-term outcomes such as life years gained (LYG) or quality-adjusted life years (QALY). Accordingly, studies were excluded if they investigated a non-screening population (e.g., CRC patients) or a specific target group (e.g., high risk population), or if they had no adequate comparator. Partial economic evaluations and studies that only reported non-comparable outcomes, such as cost per patient screened or cost per cancer detected, were also excluded. Congress abstracts, duplicate publications, and gray literature were not eligible for inclusion. The language was limited to English or German.

Two independent researchers screened the retrieved results. After all duplicate items were removed, title and abstract were assessed for eligibility. If the studies appeared suitable, full texts were obtained and reviewed based on the inclusion and exclusion criteria. Disagreement on inclusion was discussed within the research team and a final consensus was reached.

### Data extraction and analysis

Data extraction was performed by one reviewer and checked for accuracy by a second reviewer. Discrepancies were resolved within the research team, if necessary. The data was recorded in tabular form and included study population, investigated screening strategies, methodological approach and structure, perspective, time horizon, adherence rate, discount rate, and methods for sensitivity analysis. Discounted cost and effectiveness measures were extracted per person for each CRC screening strategy, including only base case values. The incremental costs and effectiveness (LYG or QALY gained) of other commonly analyzed strategies compared to sigmoidoscopy were calculated. If a strategy was equally or more costly and less effective, it was considered as dominated. Similarly, if a strategy was less costly and equally or more effective, that strategy was referred to as cost-saving. For non-dominated comparisons, the incremental cost-effectiveness ratio (ICER) was derived, stating the incremental cost per LYG or per QALY gained. Alternatively, ICERs were extracted when reported in the study.


$$ICER=\frac{{Cost\;}_{Sigmoidoscopy}-{Cost\;}_{Comparator}}{{Effectiveness\;}_{Sigmoidoscopy}-{Effectiveness\;}_{Comparator}}$$

### Cost conversion

To improve the comparability of the studies, all cost estimates were adjusted to 2019 US-$ using country-specific consumer price indices (CPI). If a study did not state the base year for costs, 2 years prior to publication was assumed as the base year. When necessary, local currency units were subsequently converted to US-$ using Purchasing Power Parity (PPP) conversion factors [32, 33]. CPI and PPP data were obtained from the World Bank Database [34].

### Quality assessment

The critical appraisal of the studies was undertaken by 2 independent researchers with a questionnaire by the International Society for Pharmacoeconomics and Outcomes Research (ISPOR) [30]. The questionnaire is a recommended tool for assessing the relevance and credibility of modeling studies based on the 7 main dimensions: validation, design, data, analysis, reporting, interpretation, and conflict of interest. Each domain consists of 1 or more questions and can be judged as strength, neutral or weakness depending on the responses. If the study demonstrated convincing credibility in all sub-questions, the domain was rated as a strength. If there were minor concerns but they did not limit the overall credibility, the domain was rated as neutral. In the case of issues that were a potential constraint on credibility, the domain represented a weakness. Disagreement between the reviewers was resolved by discussion within the research team. Based on the domain ratings, the credibility of each study was assessed as sufficient or insufficient for the review.

## Results

**Fig 1:**
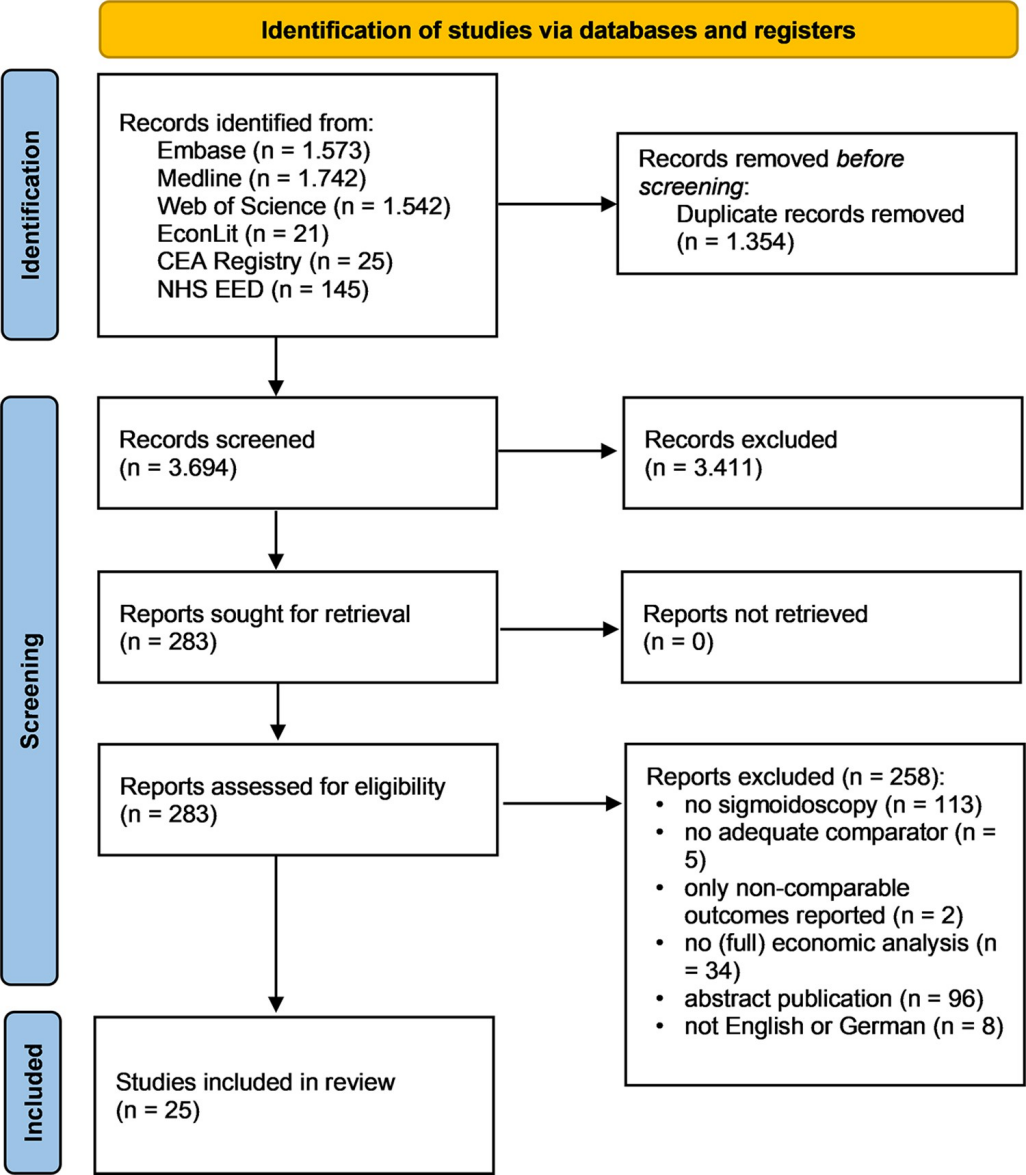
Flow diagram of study selection.

The database search yielded 5.048 records (Fig 1). After removing duplicates, 283 full-text articles were assessed for eligibility. The majority of articles were excluded because sigmoidoscopy was not evaluated or no (full) economic analysis was conducted. Other reasons included lack of an adequate comparator group, non-comparable outcomes according to the exclusion criteria, publication of an abstract with no full article, and language other than English or German. For articles excluded after full-text review, see S2 Table in the supplement. Ultimately, 25 articles could be included in the systematic review.

### 1.1 Study characteristics

The majority of the included studies were conducted in the USA [35–46], and 2 in Canada [47, 48] (Table 1). In 7 models investigated CRC screening in European countries, namely France [49, 50], UK [51, 52], Ireland [53], Italy [54], and Ukraine [55]. A total of 3 studies came from Asian countries: 2 from Hong Kong [56, 57] and 1 from Singapore [58]. A single study was conducted in Australia [59]. Fifteen studies employed a Markov Model to estimate the cost-effectiveness. The remaining 10 studies used microsimulation as a modeling approach. The majority of these 10 studies utilized at least 1 of 3 models developed by the US Cancer Intervention and Surveillance Modeling Network (CISNET) named CRC-SPIN, MISCAN and Sim-CRC [38, 42, 43, 45, 46]. US studies using microsimulation thus applied identical modeling techniques and assumptions and differed mainly in the input parameters or populations examined. To reduce the risk of ‘double counting’ and overestimating single models, studies were grouped by modeling approaches.

**Table 1:** Characteristics of the studies.

Author, Year	Country	Model Type	Model Name	Perspective	Time Horizon	Discount Rate	Outcome Measure	Screening Period	Route of Carcinogenesis	Types of precursor lesions	Consideration of the localization of lesions
Barré 2020 [50]	FR	Microsimulation	-	Societal	Lifetime	4%	QALY	50–74	100% Adenoma-Carcinoma-Sequence	Polyp or adenoma (6–9 mm; ≥ 10mm)	Consideration of proximal and distal lesions separately
Barzi 2017 [35]	US	Microsimulation	-	Societal	35 years or Lifetime	3%	LYG	50–75	100% Adenoma-Carcinoma-Sequence	Adenoma	n/a
Dan 2012 [58]	SG	Markov	-	Societal	Lifetime	3%	QALY	50–75	100% Adenoma-Carcinoma-Sequence	Polyp	n/a
Dinh 2013 [36]	US	Microsimulation	Archimedes	Societal	30 years	3%	QALY	50–75	100% Adenoma-Carcinoma-Sequence	benign polyps or adenomatous polyps	Differentiation of cecum, ascending, transverse, descending and sigmoid colon and rectum
Hassan 2011 [49]	FR	Markov	-	Third-Payer	Lifetime	3%	LYG	50–75	85% Adenoma-Carcinoma-Sequence, 15% de novo	diminutive (≤ 5 mm), medium (6–9 mm) or large (≥ 10 mm) adenomatous polyp	n/a
Heitman 2010 [47]	CA	Markov	-	Third-Payer	Lifetime	5%	QALY	50–75	100% Adenoma-Carcinoma-Sequence	nonadvanced or advanced adenoma	n/a
Kingsley 2016 [37]	US	Microsimulation	-	Societal	Lifetime	3%	QALY	50–80	Adenoma-Carcinoma-Sequence, de novo^a^	high-grade (>1cm, 3 or more in number or with villous dysplastic features on histology) or low-grade adenomas (all other)	n/a
Knudsen 2010 [38]	US	Microsimulation	CRC-SPIN MISCAN Sim-CRC	Third-Payer	Lifetime	3%	LYG	65–80	100% Adenoma-Carcinoma-Sequence	small (1–5 mm), medium (6–9 mm), and large (≥10 mm) adenoma (MISCAN and Sim-CRC), continuous adenoma growth from 1-50mm (CRC-SPIN)	Differentiation of cecum, ascending, transverse, descending and sigmoid colon and rectum
Lansdorp-Vogelaar 2010 [42]	US	Microsimulation	MISCAN Sim-CRC	Third-Payer	Lifetime	3%	LYG	65–80	100% Adenoma-Carcinoma-Sequence	small (1–5 mm), medium (6–9 mm), or large (≥10 mm) adenoma	
Naber 2019 [43]	US	Microsimulation	CRC-SPIN MISCAN Sim-CRC	Third-Payer	Lifetime	3%	LYG	65–75	100% Adenoma-Carcinoma-Sequence	small (1–5 mm), medium (6–9 mm), and large (≥10 mm) adenoma (MISCAN and Sim-CRC), continuous adenoma growth from 1-50mm (CRC-SPIN)	
Van Hees 2014 [45]	US	Microsimulation	MISCAN	Societal	Lifetime	3%	QALY	76–90	100% Adenoma-Carcinoma-Sequence	small (1–5 mm), medium (6–9 mm), or large (≥10 mm) adenoma	
Vanness 2011 [46]	US	Microsimulation	CRC-SPIN MISCAN Sim-CRC	Third-Payer	Lifetime	3%	LYG	50–80	100% Adenoma-Carcinoma-Sequence	small (1–5 mm), medium (6–9 mm), and large (≥10 mm) adenoma (MISCAN and Sim-CRC), continuous adenoma growth from 1-50mm (CRC-SPIN)	
Ladabaum 2013 [39]	US	Markov	-	Third-Payer	Lifetime	3%	QALY	50–80	85% Adenoma-Carcinoma-Sequence, 15% de novo	small (<10 mm) or large (≥10 mm) adenomatous polyp	Differentiation of fractions of lesions within reach of endoscope
Ladabaum 2018 [40]	US	Markov	-	Third-Payer	Lifetime	3%	QALY	50–80	85% Adenoma-Carcinoma-Sequence, 15% de novo	small (<10 mm) or large (≥10 mm) adenomatous polyp	
Ladabaum 2019 [41]	US	Markov	-	Third-Payer	Lifetime	3%	QALY	45–75	85% Adenoma-Carcinoma-Sequence, 15% de novo	small (<10 mm) or large (≥10 mm) adenomatous polyp	
Sharaf 2013 [44]	US	Markov	-	Third-Payer	Lifetime	3%	QALY	50–80	85% Adenoma-Carcinoma-Sequence, 15% de novo	small (<10 mm) or large (≥10 mm) adenomatous polyp	
Lam 2015 [56]	HK	Markov	-	?	25 years	n/a	LYG, QALY	50–75	100% Adenoma-Carcinoma-Sequence	low-risk or high-risk polyps	n/a
Lee 2010 [51]	UK	Markov	-	Third-Payer	Lifetime	3.5%	LYG, QALY	60–69	100% Adenoma-Carcinoma-Sequence	low-risk (6–9 mm) or high-risk polyps (≥10 mm)	Consideration of proximal and distal lesions separately
Lew 2018 [59]	AU	Microsimulation	Policy1-Bowel	Third-Payer	Lifetime	5%	LYG	50–74	85% Adenoma-Carcinoma-Sequence, 15% Serrated Pathway	diminutive (<6mm), small (≥6mm) or large (≥10mm) adenoma; Small (<10mm) or large (≥10mm) hyperplastic polyp; small (<10mm) or large (≥10mm) sessile serrated adenoma	Differentiation of cecum, ascending, transverse, descending and sigmoid colon and rectum
Senore 2019 [54]	IT	Markov	-	Third-Payer	12 years	3%	LYG	58–70	no natural history model	n/a	n/a
Sharp 2012 [53]	IE	Markov	-	Third-Payer	Lifetime	4%	QALY	55–74	86% Adenoma-Carcinoma-Sequence, 14% de novo	low-risk (<10 mm) or higher-risk (≥10 mm) adenomas	Consideration of proximal and distal lesions separately
Telford 2010 [48]	CA	Markov	-	Third-Payer	Lifetime	5%	QALY	50–75	100% Adenoma-Carcinoma-Sequence	low-risk polyp or advanced adenoma	Differentiation of fractions of lesions within reach of endoscope
Melnitchouk 2018 [55]	UA	Markov	-	Third-Payer	Lifetime	3%	QALY	50–75	100% Adenoma-Carcinoma-Sequence	low-risk or high-risk polyps	
Whyte 2012 [52]	UK	Markov	-	Third-Payer	Lifetime	3.5%	LYG, QALY	55–74	Adenoma-Carcinoma-Sequence, de novo^a^	low-risk or high-risk adenomas	n/a
Wong 2016 [57]	HK	Markov	-	?	Lifetime	3%	LYG	50–70	no natural history model	n/a	n/a

FR: France; US: United States of America; SG: Singapore; CA: Canada; HK: Hong Kong; UK: United Kingdom; AU: Australia; IT: Italy; IE: Ireland; UA: Ukraine

? Unclear

LYG: life years gained

QALY: quality-adjusted life years

n/a: not applicable

^a^ distribution unclear

In 6 studies, the analyses were conducted from a societal perspective [36, 37, 45, 46, 50, 58]. The other studies took a third-party payer perspective, with the exception of 2 studies where the perspective was not declared and remained unclear [56, 57]. Almost all studies followed the modeled cohort for a lifetime to capture the long-term effects of CRC screening. Shorter time horizons varied between 12 and 35 years [35, 36, 54, 56]. The discount rate was between 3% and 5% among all studies that reported discounting. QALY gained were the most frequently analyzed outcome, described by 13 studies [36, 37, 39–41, 44, 45, 47, 48, 50, 53, 55, 58]. A further 9 studies reported LYG [35, 38, 42, 43, 46, 49, 54, 57, 59]. Both LYG and QALY were reported in 3 studies [51, 52, 56]. The screening period, meaning the time frame where screening measures were actively performed, covered at least the period from 55 to 70 years of age in 18 studies. Screening started as early as 45 years of age in 1 study [41], while the earliest screening age in another study was as high as 76 [45], with 50 being the most common starting age. Most studies had a screening period of either 26 or 31 years.

### Structural assumptions

When comparing the validity of the models, some structural aspects are of particular importance: the displayed carcinogenesis and the type and localization of lesions modeled. These aspects can lead to crucial differences in CRC detection between endoscopic procedures and stool tests, but also between colonoscopy and sigmoidoscopy.

In 2 studies, the natural history of CRC was not modeled [52, 54] (Table 1). All other models depicted the adenoma-carcinoma sequence. De novo carcinoma without previous adenomatous lesions were additionally included in 8 studies [37, 39–41, 44, 49, 52, 53]. Only 1 microsimulation also modeled the serrated pathway and therefore explicitly included sessile serrated adenoma as a precursor lesion of CRC [59]. Moreover, modeling of lesions was performed differently across models. Most studies classified either 2 (14 studies) [36, 37, 39–41, 44, 47, 48, 50–53, 55, 56] or 3 types of adenomas (7 studies) [38, 42, 43, 45, 46, 49, 59]. Two studies modelled only 1 stage of adenoma [35, 58]. While 11 studies differentiated adenomas solely by size [38–46, 49, 59], 9 studies composed risk types, some of which also included histology [36, 37, 47, 48, 51–53, 55, 56]. The CRC-SPIN microsimulation modeled adenoma growth as continuous growth rather than set health states. 16 studies that modeled the natural history explicitly addressed the localization of lesions. A separate modeling of all sections of the intestine was only conducted in microsimulations [36, 38, 42, 43, 45, 46, 59]. Two Markov models and 1 microsimulation included distal and proximal adenomas as different health states [50, 51, 53]. An indirect approach to localization using different fractions of lesions within reach of the respective endoscope was performed in 6 articles, which are primarily adaptions of 2 modeling approaches [39–41, 44, 48, 55].

### Quality assessment

**Fig 2:**
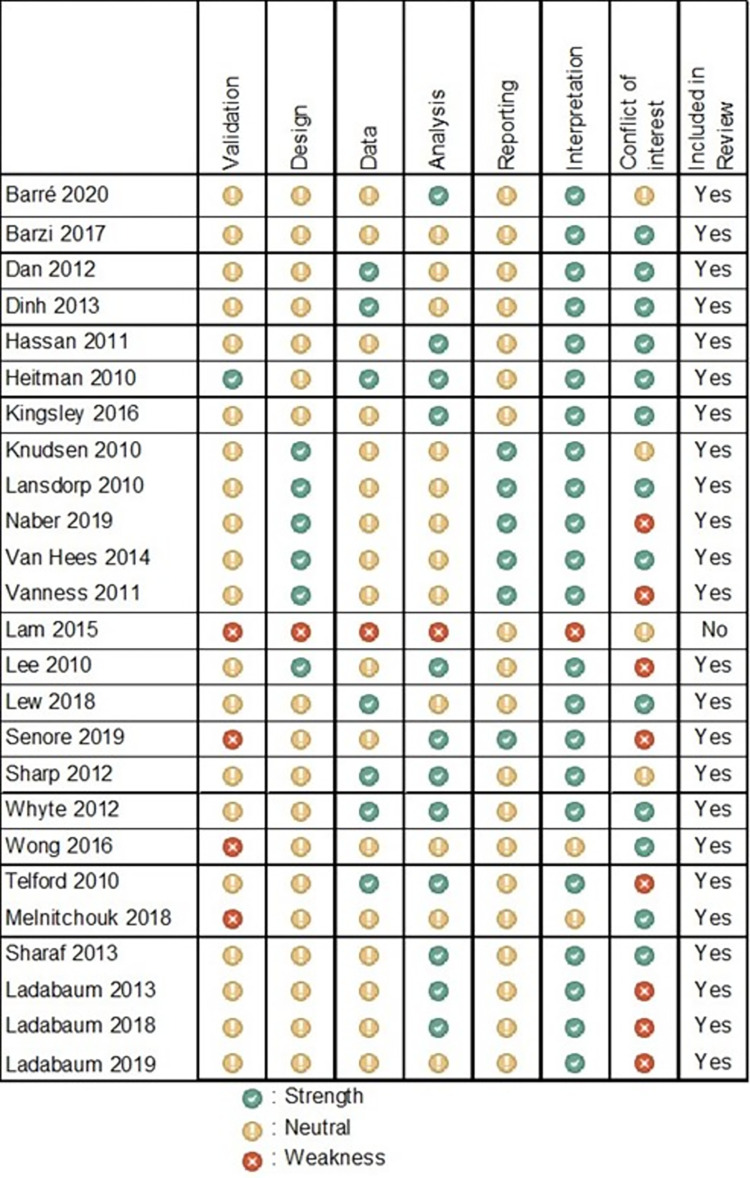
Results of the quality assessment of the retrieved modelling approaches based on the ISPOR “Questionnaire to assess relevance and credibility of modeling studies for informing health care decision making” [30].

The overall credibility of the studies was sufficient (Fig 2). Only the study by Lam et al. [56] was excluded from the result synthesis due to severe weaknesses in almost all domains. The most critical domain across the models was validation, even though most authors ensured sufficient external validity. In 3 studies, serious concerns for validity were present, as it was unclear whether the results of the models are coherent with independent data [54, 55, 57]. Validation issues were mostly based on the lack of internal validity, as almost no model documented technical verification of equations and debugging exercises ensuring all assumptions were correctly implemented in code. While the model designs and included data were generally appropriate for the course of CRC, limitations resulted from the often unclear process of model development and data identification. Analysis was assessed as a strength in 11 studies [37, 39, 40, 44, 47, 49–54] and as neutral in 12 studies [35, 36, 38, 41–43, 45, 46, 55, 57–59], due for example to lack of probabilistic sensitivity analysis. Reporting and interpretation was predominantly satisfactory. Only the technical documentation was absent in the majority of models, making replication of the results difficult. Eight studies raised the distinct possibility of a conflict of interest [39–41, 43, 46, 48, 51, 54].

### 1.2 Cost-effectiveness results

In the models, sigmoidoscopy was explored within different screening strategies. As a single-intervention strategy, a 5-year interval was the most common (13 studies) [35–40, 42, 43, 47–49, 57, 58]. A number of 5 studies investigated sigmoidoscopy at a 10-year interval [49–52, 59] and 7 as a once-only measure [44, 45, 52–54, 58, 59]. Cost-effectiveness results of these other intervals are displayed in the appendix (S1 File). In addition, sigmoidoscopy was assessed in combination with stool testing (8 studies) [35, 38–40, 46, 48, 55, 58]. Models with a societal perspective did not differ in the overall results from third-party payer models.

### Sigmoidoscopy every 5 years

Compared to ‘no screening’, 5-yearly sigmoidoscopy was in the majority of studies less costly and more effective, and therefore cost-saving (Table 2). Sigmoidoscopy always yielded higher QALY or LYG than no screening, with the highest ICER being $50,027.19 per LYG [57]. Sigmoidoscopy was in all studies less effective than the gold-standard endoscopic procedure colonoscopy and in 5 studies associated with higher cost per patient [35, 36, 38, 43, 47]. ICER were mostly below $67,000, favoring colonoscopy. One study reported significantly higher incremental cost of approximately $107,365.00 per LYG with colonoscopy compared to sigmoidoscopy [49]. Regarding stool tests, the results were less consistent. Annual guaiac-based FOBT dominated sigmoidoscopy in 7 comparisons within 4 studies [38, 40, 43, 48], but in 6 cases sigmoidoscopy was associated with higher effectiveness and incremental cost below $26,000 per LYG or QALY [38, 47, 49]. Most models showed that modern annual FIT was more effective and less costly than sigmoidoscopy [36, 38–40, 43, 47]. In 4 non-dominated comparisons with FIT, incremental cost varied between $20,182.42 and $194,487.07 per LYG [43, 47, 49, 58]. Only the studies by Barzi et al. [35] and Telford et al. [48] showed that sigmoidoscopy was cost-saving compared to both FOBT and FIT. Biennial FOBT or FIT was evaluated in only a few studies with mixed results when compared to sigmoidoscopy (S1 File). Overall, there was a tendency for sigmoidoscopy to be favored over low-sensitivity FOBT but dominated by higher sensitivity FOBT and FIT. Most of the variability of the stool test results was therefore attributable to differences in sensitivity assumptions.

**Table 2:** Incremental cost, life years gained, quality-adjusted life years and incremental cost-effectiveness ratios of 5-yearly sigmoidoscopy compared with commonly used screening measures (in 2019 $).

				5-yearly sigmoidoscopy															
Author, Year	Country	Model Name	Screening Period	vs. no screening				vs. 10-yearly colonoscopy				vs. annual FOBT				vs. annual FIT			
				∆Cost	LYG	∆QALY	ICER	∆Cost	LYG	∆QALY	ICER	∆Cost	LYG	∆QALY	ICER	∆Cost	LYG	∆QALY	ICER
Barzi 2017 [35]	US	Markov	50–75	-291.234	0.016		**CS**	306.335	-0.006		**D**	-20.494	0.006		**CS**	-170.426	0.010		**CS**
Dan 2012 [58]	SG	Markov	50–75	249.736		0.005	49,947.163	-474.136		-0.012	39,511.343	-	-	-	-	97.723		0.001	97,722.711
Dinh 2013 [36]	US	Archimedes	50–75	-1,265.065		0.071	**CS**	42.208		-0.044	**D**	-	-	-	-	406.837		-0.025	**D**
Hassan 2011 [49]	FR	Markov	50–75	186.815	0.045		4,135.822	-254.455	-0.002		107,364.996	86.966	0.005		16,628.231	-78.913	-0.004		20,182.422
Heitman 2010 [47]	CA	Markov	50–75	515.069		0.036	14,307.482	231.923		-0.005	**D**	96.753		0.020	4,837.668^c^	367.094		0.009	40,788.182^c^
												254.689		0.024	10,612.041^d^	611.823		-0.009	**D** ^e^
																368.516		-0.011	**D** ^d^
Kingsley 2016 [37]	US	Markov	50–80	-204.138		0.062	**CS**	-556.939		-0.038	14,503.624	-	-	-	-	362.164		-0.027	**D**
Knudsen 2010^a^ [38]	US	CRC-SPIN	65–80	-321.818	0.076		**CS**	143.030	-0.030		**D**	221.944	0.012		18,808.785^f^	173.856	-0.009		**D**
												288.527	-0.012		**D** ^g^				
Naber 2019 [43]	US	CRC-SPIN	65–75	-853.166	0.071		**CS**	658.127	-0.037		**D**	754.082	-0.019		**D**	651.869	-0.018		**D**
Knudsen 2010^a^ [38]	US	MISCAN	65–80	133.166	0.075		1,775.549	-102.341	-0.012		8,747.067	235.507	0.009		25,323.321^f^	56.719	-0.005		**D**
												131.933	-0.006		**D** ^g^				
Naber 2019 [43]	US	MISCAN	65–75	-91.783	0.089		**CS**	33.376	-0.013		**D**	290.994	0.002		126,519.292	330.628	0.002		194,487.067
Knudsen 2010^a^ [38]	US	Sim-CRC	65–80	-245.371	0.065		**CS**	-6.165	-0.029		215.563	106.040	0.005		20,007.500^f^	64.117	-0.015		**D**
												155.361	-0.016		**D** ^g^				
Naber 2019 [43]	US	Sim-CRC	65–75	-503.764	0.080		**CS**	205.469	-0.023		**D**	489.163	-0.012		**D**	492.292	-0.012		**D**
Ladabaum 2013^b^ [39]	US	Markov	50–80	-239.178		0.069	**CS**	-473.667		-0.007	66,713.599	242.695		0.002	121,347.742	344.698		-0.008	**D**
Ladabaum 2018 [40]	US	Markov		775.778		0.050	15,577.862	-576.769		-0.026	22,355.369	1,340.629		-0.017	**D**	1,064.162		-0.027	**D**
Telford 2010 [48]	CA	Markov	50–75	844.810		0.074	11,416.350	-241.790		-0.046	5,256.313	-75.742		0.005	**CS** ^c^	-107.786		15.270	**CS**
												10.196		-0.019	**D** ^d^				
Wong 2016 [57]	HK	Markov	50–70	1,134.617	0.023		50,027.187	-502.169	-0.020		25,647.035	-	-	-	-	-	-	-	-

FR: France; US: United States of America; SG: Singapore; CA: Canada; HK: Hong Kong; UK: United Kingdom; AU: Australia; IT: Italy; IE: Ireland; UA: Ukraine

CS: cost saving, when a strategy was less costly and equally or more effective

D: dominant, when a strategy was equally or more costly and less effective

LYG: life years gained

QALY: quality-adjusted life years

ICER: incremental cost-effectiveness ratio

n/a: not applicable

FOBT: guaiac-based fecal occult blood test

FIT: fecal immunochemical tests

^a^ Identifical model and input data to Lansdorp-Vogelaar 2010, therefore only Knudsen 2010 reported

^b^ Identical model and input data to Sharaf & Ladabaum 2013, therefore only Ladabaum 2013 reported

^c^ Low-Sensitivity Test

^d^ High-Sensitivity Test

^e^ Mid-Sensitivity Test

^f^ Hemoccult II Test

^g^ Hemoccult SENSA

In 10 different models, sigmoidoscopy was analyzed alongside newer screening strategies, such as DNA testing [35, 37, 39, 42, 43, 47, 48, 58], CT-colonography [35, 38, 47, 48, 58] or capsule endoscopy [49] (Table 3). DNA (stool) tests were conducted at an interval of either 2 [35, 39], 3 [37, 42, 43, 47, 48] or 5 years [42, 58]. While sigmoidoscopy was cost-saving in most comparisons, some studies came to the opposite conclusion, with sigmoidoscopy being associated with a smaller effect than DNA testing. However, ICERs for DNA testing were rather high, between $61,310.88 and $200,018.32 per QALY or per LYG, respectively [37, 42, 43, 58]. Compared to CT-colonography, 5-yearly sigmoidoscopy was associated with lower cost, but also lower effectiveness. The lowest incremental costs were $21,407.95 per LYG [38]. Barzi et al. analyzed the only modeling approach where sigmoidoscopy was dominated by CT-colonography [35]. This was also the only study where the interval of the comparator intervention was 10 years. In all other studies examining this strategy as a comparator, both CT-colonography and sigmoidoscopy were conducted every 5 years. Only Hassan and colleagues included capsule endoscopy, generating no conclusive results [49].

**Table 3:** Incremental cost, life years gained, quality-adjusted life years and incremental cost-effectiveness ratios of 5-yearly sigmoidoscopy compared with newer screening measures (in 2019 $).

				5-yearly sigmoidoscopy											
Author, Year	Country	Model Name	Screening Period	vs. DNA Test				vs. CT-Colonography				vs. Capsule Endoscopy			
				∆Cost	LYG	∆QALY	ICER	∆Cost	LYG	∆QALY	ICER	∆Cost	LYG	∆QALY	ICER
Barzi 2017 [35]	US	Markov	50–75	-1,095.904	0.002		**CS** ^a^	89.528	-0.004		**D** ^h^	-	-	-	-
Dan 2012 [58]	SG	Markov	50–75	-161.665		-0.002	80,832.366^b^	-796.259		-0.012	66,354.927^b^	-	-	-	-
Hassan 2011 [49]	FR	Markov	50–75	-	-	-	-	-	-	-	-	-644.995	-0.004		167,967.503^b^
				-	-	-	-	-	-	-	-	-268.144	0.002		**CS** ^h^
Heitman 2010 [47]	CA	Markov	50–75	-650.240		0.026	**CS** ^c^	-207.735		-0.005	41,547.032^b^	-	-	-	-
				-324.408		0.013	**CS** ^d^	-	-	-	-	-	-	-	-
Kingsley 2016 [37]	US	Markov	50–80	-1,741.229		-0.028	61,310.867^e^	-	-	-	-	-	-	-	-
Knudsen 2010 [38]	US	CRC-SPIN	65–80	-	-	-	-	-543.762	-0.025		21,407.952^i^	-	-	-	-
				-	-	-	-	-563.490	-0.022		25,382.446^i^	-	-	-	-
Naber 2019 [43]	US	CRC-SPIN	65–75	-810.404	-0.008		95,341.61^e^					-	-	-	-
Knudsen 2010 [38]	US	MISCAN	65–80	-	-	-	-	-797.764	-0.010		77,452.831^i^	-	-	-	-
				-	-	-	-	-821.192	-0.006		130,347.864^i^	-	-	-	-
Lansdorp-Vogelaar 2010 [42]	US	MISCAN	65–80	-1,048.437	0.006		**CS** ^**e**^	-	-	-	-	-	-	-	-
				-690.245	0.016		**CS** ^**b**^	-	-	-	-	-	-	-	-
Naber 2019 [43]	US	MISCAN	65–75	-1054.463	0.007		**CS** ^**e**^	-	-	-	-	-	-	-	-
Knudsen 2010 [38]	US	Sim-CRC	65–80	-	-	-	**-**	-623.908	-0.027		23,280.160^i^	-	-	-	-
				-	-	-	**-**	-662.132	-0.022		30,096.906^i^	-	-	-	-
Lansdorp-Vogelaar 2010 [42]	US	Sim-CRC	65–80	-1100.101	-0.006		200,018.317^e^	-	-	-	-	-	-	-	-
				-770.884	0.007		**CS** ^**b**^	-	-	-	-	-	-	-	-
Naber 2019 [43]	US	Sim-CRC	65–75	-948.078	-0.008		121,548.518^e^	-	-	-	-	-	-	-	-
Ladabaum 2013 [39]	US	Markov	50–80	-964.920		0.015	**CS** ^f^	-	-	-	-	-	-	-	-
				-848.848		0.007	**CS** ^g^	-	-	-	-	-	-	-	-
Telford 2010 [48]	CA	Markov	50–75	-952.596		0.016	**CS** ^**e**^	-1124.471		0.003	**CS** ^b^	-	-	-	-

FR: France; US: United States of America; SG: Singapore; CA: Canada; HK: Hong Kong; UK: United Kingdom; AU: Australia; IT: Italy; IE: Ireland; UA: Ukraine

CS: cost saving, when a strategy was less costly and equally or more effective

D: dominant, when a strategy was equally or more costly and less effective

LYG: life years gained

QALY: quality-adjusted life years

ICER: incremental cost-effectiveness ratio

n/a: not applicable

^a^ Comparator intervention biennial

^b^ Comparator intervention every 5 years

^c^ Comparator is first generation fecal DNA test every 3 years

^d^ Comparator is second generation fecal DNA test every 3 years

^e^ Comparator intervention triennial

^f^ Compartor intervention is biennial ^m^SEPT9-2well test

^g^ Compartor intervention is biennial ^m^SEPT9-3well test

^h^ Comparator intervention every 10 years

^i^ Different performance assumptions of CT-colonography

### Sigmoidoscopy in combination with stool tests

Similarly to sigmoidoscopy alone, combinatorial strategies were also more effective or even cost-saving compared to no screening (S1 File). Seven models evaluated 5-yearly sigmoidoscopy in combination with annual guaiac-based FOBT [35, 38–40, 46, 48, 55, 58] (Table 4). When comparing this strategy to the standard colonoscopy, results were mixed. Although sigmoidoscopy was cost-saving in 5 cases compared to colonoscopy [38–40, 46], it was dominated in 3 studies [35, 38, 55] and both less expensive and less effective in the remaining models [38, 46, 48]. The highest incremental cost was $78,776.30 per LYG [38]. When comparing the combinatorial strategy of sigmoidoscopy plus annual FOBT with the respective strategies alone, the evaluations showed a clear improvement of LYG or QALYs. In 4 models, the addition of FOBT annually resulted in cost-savings while gaining LYG or QALY per patient compared to sigmoidoscopy alone [38, 40]. Compared with FOBT alone, the combination with sigmoidoscopy resulted in incremental cost of $143.37 to $88,088.28 per LYG or QALY [38, 40].

**Table 4:** Incremental cost, life years gained, quality-adjusted life years and incremental cost-effectiveness ratios of 5-yearly sigmoidoscopy combined with annual stool tests compared with single measure strategies (in 2019 $).

				5-yearly sigmoidoscopy + annual FOBT											
Author, Year	Country	Model Name	Screening Period	vs. 10-yearly colonoscopy				vs. 5-yearly sigmoidoscopy				vs. annual FOBT			
				∆Cost	LYG	∆QALY	ICER	∆Cost	LYG	∆QALY	ICER	∆Cost	LYG	∆QALY	ICER
Barzi 2017 [35]	US	Markov	50–75	333.302	-0.010		**D**	26.966	-0.004		**D**	6.472	0.002		3,235.938
Dan 2012 [58]	SG	Markov	50–75	-	-	-	-	-	-	-	-	-	-	-	-
Knudsen 2010^a^ [38]	US	CRC-SPIN	65–80	69.049	-0.013		**D** ^b^	-73.981	0.017		**CS** ^b^	147.962	0.029		5,119.808^b^
				82.612	-0.006		**D** ^c^	-60.418	0.024		**CS** ^c^	228.109	0.013		18,103.870^c^
Vanness 2011 [46]	US	CRC-SPIN	50–80	-109.739	-0.004		27,434.703	-	-	-	**-**	-	-	-	-
Knudsen 2010^a^ [38]	US	MISCAN	65–80	-141.797	-0.002		78,776.299^b^	-39.457	0.010		**CS** ^b^	196.050	0.019		10,210.945^b^
				-56.719	0.001		**CS** ^c^	45.622	0.013		3509.366^c^	177.555	0.007		25,732.598^c^
Vanness 2011 [46]	US	MISCAN	50–80	-41.923	0.004		**CS**	-	-	-	**-**				
Knudsen 2010^a^ [38]	US	Sim-CRC	65–80	-108.506	-0.008		13,395.777^b^	-102.341	0.021		**CS** ^b^	3.699	0.026		143.374^b^
				-27.126	-0.001		20,866.498^c^	-20.961	0.027		**CS** ^c^	134.399	0.011		11,789.405^c^
Vanness 2011^d^ [46]	US	Sim-CRC	50–80	-219.478	0.001		**CS**	-	-	-	**-**	-	-	-	-
Ladabaum 2013 [39]	US	Markov	50–80	-182.901		0.003	**CS**	290.766		0.010	27,691.962	533.461		0.012	42,676.887
Ladabaum 2018 [40]	US	Markov		-816.294		0.003	**CS**	-239.526		0.029	**CS**	1,101.104		0.012	88,088.284
Melnitchouk 2018 [55]	UA	Markov	50–75	117.117		-0.007	**D**	-	-	-	-	-	-	-	-
Telford 2010 [48]	CA	Markov	50–75	-864.094		-0.046	18,784.645	340.837		0.032	10,651.159	351.033		0.013	27002.544^e^
				**5-yearly sigmoidoscopy + annual FIT**											
Author, Year	Country	Model Name	Screening Period	vs. 10-yearly colonoscopy				vs. 5-yearly sigmoidoscopy				vs. annual FIT			
				∆Cost	LYG	∆QALY	ICER	∆Cost	LYG	∆QALY	ICER	∆Cost	LYG	∆QALY	ICER
Barzi 2017 [35]	US	Markov	50–75	514.514	-0.013		**D**	208.179	-0.007		**D**	37.753	0.003		12,584.202
Dan 2012 [58]	SG	Markov	50–75	-360.730		-0.010	36,072.951	113.407		0.002	56,703.301	211.129		0.003	70,376.438
Knudsen 2010^a^ [38]	US	CRC-SPIN	65–80	208.380	-0.006		**D**	65.350	0.023		2,792.738	239.206	0.015		16,496.962
Vanness 2011 [46]	US	CRC-SPIN	50–80	19.728	-0.004		**D**	-	-	-	**-**	-	-	-	**-**
Knudsen 2010^a^ [38]	US	MISCAN	65–80	143.030	0.001		102,164.542	245.371	0.013		18,730.614	302.090	0.008		37,761.248
Vanness 2011 [46]	US	MISCAN	50–80	56.719	0.004		14,179.734	-	-	-	-	-	-	-	-
Knudsen 2010^a^ [38]	US	Sim-CRC	65–80	86.311	-0.002		**D**	92.477	0.027		3,412.418	156.594	0.013		12,527.487
Vanness 2011^d^ [46]	US	Sim-CRC	50–80	-97.409	0.001		**CS**	-	-	-	**-**	-	-	-	-
Ladabaum 2013 [39]	US	Markov	50–80	-179.384		0.006	**CS**	294.283		0.013	23,355.788	638.981		0.004	152,138.301
Ladabaum 2018 [40]	US	Markov		-551.743		0.006	**CS**	25.025		0.031	796.977	1,089.187		0.004	259,330.202

FR: France; US: United States of America; SG: Singapore; CA: Canada; HK: Hong Kong; UK: United Kingdom; AU: Australia; IT: Italy; IE: Ireland; UA: Ukraine

CS: cost saving, when a strategy was less costly and equally or more effective

D: dominant, when a strategy was equally or more costly and less effective

LYG: life years gained

QALY: quality-adjusted life years

ICER: incremental cost-effectiveness ratio

FOBT: guaiac-based fecal occult blood test

FIT: fecal immunochemical tests

^a^ Identifical model and input data to Lansdorp-Vogelaar 2010, therefore only Knudsen 2010 reported

^b^ Hemoccult II Test

^c^ Hemoccult SENSA

^d^ Sigmoidoscopy without biopsy

^e^ High sensitivity FOBT

Five-yearly sigmoidoscopy in combination with annual FIT was evaluated in 6 models [35, 38–40, 46, 58]. Results of the comparison of this strategy with colonoscopy were similar to those of the combination with FOBT. Incremental cost varied even within 1 modeling approach between $14,179.73 and $102,164.54 [38, 46]. Compared with 5-yearly sigmoidoscopy, the combination with FIT was not cost-saving, but in almost all models yielded higher effectiveness levels with ICERs between $796.98 and $56,703.30 [38–40, 58]. Sigmoidoscopy in conjunction with FIT was more expensive, but also more effective than FIT alone. The incremental cost ranged between $12,527.49 per LYG and $259,330.20 per QALY [38, 40].

The evaluation of 5-yearly sigmoidoscopy plus biennial or triennial stool tests was not significantly different from combinations with annual FOBT and FIT, although only 3 models considered these strategies (S1 File).

### 1.3 Contributing factors and drivers of cost-effectiveness

In comprehensive modeling approaches, certain parameters have greater influence on cost-effectiveness results, which is estimated in sensitivity analyses (SA). The most often investigated parameters in the included articles are displayed in Table 5. All models conducted deterministic sensitivity analysis (DSA), including one-way, multi-way or scenario SA. Additionally, in about half of the studies, probabilistic sensitivity analysis (PSA) was performed to represent uncertainty in parameter estimation.

**Table 5:** Influential variables and indicators.

				Test characteristics of sigmoidoscopy			Adherence		Cost Components					Effect indicators	
Author, Year	Country	Model Type	PSA	Base Case Sensitivity for precursor lesions	Base Case Sensitivity for CRC	Base Case Specificity	General Adherence rates	Adherence rate specific to sigmoidoscopy	Cost of sigmoidoscopy (2019 US-$)	Cost of cancer care (2019 US-$)				Basline CRC risk	CRC incidence reduction attributed to 5-yearly sigmoidoscopy
										Stage I Localized CRC	Stage II	Stage III Regional CRC	Stage IV Distant CRC		
Barré 2020 [50]	FR	Microsimulation	✓	Adenoma ≤ 6 mm: 45% Adenoma > 6 mm: 90%	95%	95%	25%-65%	25%	71.24	9,663.53	12,440.73	17,466.67	20,843.50	n/a	n/a
Barzi 2017 [35]	US	Microsimulation		75%	75%	92%	46%-63%	63%	177.08	38,905.68	38,905.68	38,905.68	101,102.56^c^	4.50%	11.00%
Dan 2012 [58]	SG	Markov		65%	95%	60%	50%	-	241.29	12,064.53	24,129.06	28,954.88	21,716.16	2.03%	28%
Dinh 2013 [36]	US	Archimedes		n/a	n/a	n/a	100%	-	188.76	31,425.99	43,369.02	52,878.08	69,049.14	6.00%	53.00%
Hassan 2011 [49]	FR	Markov	✓	Diminutive (≤ 5 mm) polyps: 45% Medium (6–9 mm) polyps: 65% Large (≥ 10 mm) polyps: 65%	65%	90%	40%	-	368.80	15,628.05	-	24,699.05	29,474.11	5.36%	27.00%
Heitman 2010 [47]	CA	Markov	✓	Nonadvanced adenoma: 65% Advanced adenoma: 75%	75%	100%	63%-68%	-	924.85	35,640.81	51,425.83	137,685.72	190,680.95	4.86%	58.08%
Kingsley 2016 [37]	US	Microsimulation	✓	Low Grade polyp: 85% High Grade polyp: 90%	95%	92%	65%-95%	-	200.62–252.43	n/a	n/a	n/a	n/a	n/a	n/a
Knudsen 2010^a^ [38]	US	CRC-SPIN		Small (1-5mm) adenoma: 75% Medium (6-9mm) adenoma: 85% Large (≥10 mm) adenoma: 95%	95%	100%	100%	-	429.09	31,425.99	43,369.02	52,878.08	69,049.14^d^	5.30%	60.38%
Naber 2019 [43]	US	CRC-SPIN				87%		-	351.20	34,850.49	49,315.72	70,193.27	102,141.11^d^	6.40%	54.69%
Knudsen 2010^a^ [38]	US	MISCAN		Small (1-5mm) adenoma: 75% Medium (6-9mm) adenoma: 85% Large (≥10 mm) adenoma: 95%	95%	100%	100%	-	429.09	31,425.99	43,369.02	52,878.08	69,049.14^d^	5.70%	47.37%
Naber 2019 [43]	US	MISCAN				92%		-	351.20	34,850.49	49,315.72	70,193.27	102,141.11^d^	6.10%	50.82%
Van Hees 2014 [45]	US	MISCAN				87%		-	611.28	40,257.55	54,031.57	65,582.17	85,370.19^d^	n/a	-0.62% - 1.2%^h^
Knudsen 2010^a^ [38]	US	Sim-CRC		Small (1-5mm) adenoma: 75% Medium (6-9mm) adenoma: 85% Large (≥10 mm) adenoma: 95%	95%	100%	100%	-	429.09	31,425.99	43,369.02	52,878.08	69,049.14^d^	6.00%	51.67%
Naber 2019 [43]	US	Sim-CRC				87%		-	351.20	34,850.49	49,315.72	70,193.27	102,141.11^d^	6.40%	56.25%
Ladabaum 2013^b^ [39]	US	Markov	✓	Small polyp: 85% Large polyp: 90%	95%	92%	100%	-	198.14	33,320.80	-	56,066.17	73,211.97	5.93%	68.23%
Ladabaum 2018 [40]	US	Markov	✓			92%		-	391.98–470.37	44,216.93	-	74,400.76	97,154.51^e^	5.93%	46.53%
										32,749.49		55,112.38	71,966.17^f^		
Ladabaum 2019 [41]	US	Markov	✓			87%		-	391.98–470.37	49,327.00	-	82,999.35	108,382.15^e^	n/a	n/a
										36,538.37		61,481.34	80,283.00^f^		
Lee 2010 [51]	UK	Markov	✓	Low-risk proximal polyps: 0% Low-risk distal polyps: 95% High-risk proximal polyps: 0% High-risk distal polyps: 88.3%	Proximal CRC: 0% Distal CRC: 88.3%	100%	60%	-	267.66	6,156.57	11,381.75	17,806.37	12,171.14	n/a	n/a
Lew 2018 [59]	AU	Policy1-Bowel		Adenoma of any size: 40.9% Adenoma >5mm: 46.2% Adenoma >10mm: 47.6%	47.90%	93.20%	40%	15%	1,847.32	56,826.56	87,114.87	136,547.54	112,997.32	0.06%	8%-52%
Melnitchouk 2018 [55]	UA	Markov		Low risk polyp: 92% High risk polyp: 97%	93%	100%	75%-84%	75%	111.54	2,788.50	-	50,193.05	111,540.10	n/a	n/a
Senore 2019 [54]	IT	Markov	✓	n/a	n/a	n/a	19%-42%	30%	109.21	11,218.27	14,240.07	20,371.73	25,452.20	n/a	32%^g^
Sharp 2012 [53]	IE	Markov	✓	Low-risk distal adenomas: 65% Intermediate / high-risk distal adenomas: 74%	90%	92%	39%-53%	39%	120.49	18,382.27	29,219.65	38,581.48	28,755.38	5.16%	n/a
Telford 2010 [48]	CA	Markov	✓	Low risk polyp: 92% Advanced adenoma: 97%	93%	100%	73%	-	281.12	20,004.52	39,465.73	51,038.17	105,282.25	n/a	63.00%
Whyte 2012 [52]	UK	Markov	✓	Low-risk adenoma: 22% High-risk adenoma: 71%	62%	100%	54%-85%	85%	162.66	10,389.30	14,294.78	19,604.12	21,440.08	n/a	n/a
Wong 2016 [57]	HK	Markov	✓	-	75%	76.60%	90%-98.9%	90%	309.83	20,032.56	20,032.56	33,066.07	86,838.83	2.38%	28.93%

FR: France; US: United States of America; SG: Singapore; CA: Canada; HK: Hong Kong; UK: United Kingdom; AU: Australia; IT: Italy; IE: Ireland; UA: Ukraine

PSA: probabilistic sensitivity analysis

^a^ Identifical model and input data to Lansdorp-Vogelaar 2010 and Vanness 2011, therefore only Knudsen 2010 reported

^b^ Identical model and input data to Sharaf & Ladabaum 2013, therefore only Ladabaum 2013 reported

^c^ Barzi only differentiates costs between curable (Stage I to III) and non-curable CRC (Stage IV)

^d^ This study originally differentiates costs between initial costs (first 12 months after diagnosis), continuing cost (after 12 months) and terminal cost (final 12 month of life). For better comparability with the other studies, only the initial costs are reported in this table.

^e^ Commercial payment rates for persons under age 65

^f^ Medicare payments for persons age 65 and older

^g^ Incidence reduction as input-parameter

^h^ negative incidence reduction due to over-diagnosis

Adherence rate as well as costs of screening measures and cancer treatment varied in most models. The adherence rate to screening varied between 19% and 100%. Perfect or near perfect conditions with uptake rates over 90% across all strategies were assumed in 6 models (11 articles) [36, 38–46, 57], and only 9 studies differentiated adherence between screening measures [35, 37, 47, 50, 52–55, 57]. Unit cost of screening measures was heterogeneous among models. The lowest cost of sigmoidoscopy was $ 71.24 [50], while the highest cost assumption was over 25-fold more costly [59]. The sources for the unit costs were based on Medicare reimbursement values between the years 2007 and 2014 in 9 studies [35–41, 43, 45]. Other costing sources used were local schemes [47–54, 57, 58] as well as expert opinions [55] or unspecified assumptions [51]. Another influential parameter was the cost of cancer treatment. In particular, the cost in higher stages differed significantly across models. For example, the cost of treating stage IV or distant CRC ranged from $12,171.14 [51] to $190,680.95 [47]. Also, the difference within a model between localized/stage I CRC and the highest stage was extremely heterogeneous, ranging from barely doubling to a 40-fold increase in cost.

Different assumptions about test performance characteristics of sigmoidoscopy influenced the cost-effectiveness results, which were investigated in several sensitivity analyses. For both sensitivity and specificity, there was a wide variation in the values assumed, with possible implications for detection rates and thus the effectiveness of the screening measures evaluated. Most models differentiated sensitivity for different types of precursor lesions as well as for CRC. For adenomas, sensitivity of sigmoidoscopy ranged from 22% to 97%, and for CRC from 48% to 95%. Specificity was mostly close to 100% with the lowest assumption being 60%.

The modeled risk reduction through screening measures indicates important information about the effectiveness of CRC screening strategies, as endoscopic measures may prevent CRC development by removing precursor lesions. While the lifetime risk for developing CRC ranged from 0.06% to 6.4% (mean 4.88%; median 5.53%), the risk reduction of sigmoidoscopy varied more significantly. While in 1 study, the use of sigmoidoscopy led up to a 68.23% lower risk of developing CRC [39], another study reported an increased CRC risk due to overdiagnosis in the elderly (-0.62% risk reduction) [45].

Although SAs mostly supported the results of the base-case analysis, the effect on sigmoidoscopy was not always considered or reported. Given the fact that the models were highly heterogeneous, it was not possible to estimate the effect of the characteristics on the results. Nonetheless, the consideration of the most influential parameters revealed potential rationales for variations in the results.

## Discussion

This systematic review included the results of 25 studies. Sigmoidoscopy, both as a stand-alone strategy and in combination with stool testing, was cost-effective and in some cases cost-saving compared to no screening. In a direct comparison of effectiveness and cost to other commonly used screening strategies, sigmoidoscopy tended to be inferior to colonoscopy and FIT and had a more favorable cost-effectiveness ratio only when compared with the low-sensitivity gFOBT. At the same time, sigmoidoscopy combined with annual stool testing was more effective and less costly than the respective individual strategies. Adherence, cost assumptions, and test characteristics had the highest impact on cost-effectiveness.

Other systematic reviews have come to similar conclusions. For example, several reviews did not identify sigmoidoscopy as the optimal (single) strategy in any case [24–28]. Some of the included studies evaluated stool testing as the preferred strategy over endoscopic procedures such as colonoscopy or sigmoidoscopy. However, this is only true when considering single strategies. For the first time, the present review also focused on combined strategies of stool testing and sigmoidoscopy, demonstrating a higher benefit at low additional cost compared to the single application of the respective screening measures. Almost all studies that considered combinations of stool test and sigmoidoscopy were conducted in the USA. Combinatorial strategies have so far been of minor interest in systematic reviews of cost-effectiveness models. Only Patel and Kilgore identified a superiority of the combination of sigmoidoscopy and stool testing compared to the respective individual strategies when exclusively considering US cost-effectiveness modeling studies from 2007–2014 [27]. It is unclear whether a combination of colonoscopy with stool tests would similarly influence cost-effectiveness compared to colonoscopy alone. No evidence is available to show whether combining sigmoidoscopy with FIT or gFOBT leads to a significantly greater reduction in incidence and mortality than sigmoidoscopy alone, as RCTs have primarily examined single sigmoidoscopy [60].

Although the overall quality of the studies was satisfactory, in some cases there was no comprehensive validation of the results, which particularly included internal validation. Consequently, the results could not be fully assessed. Furthermore, the process of data identification was not presented transparently, which gave rise to the suspicion of cherry-picking of input studies. A narrative review of the quality of CRC modeling studies revealed similar deficiencies: for studies from a period of 1999–2014, the main criticisms of the authors were the oversimplification of carcinogenesis and insufficient, as well as outdated, evidence as input to the models [18]. In an assessment of the reporting quality of recent modeling studies using the Consolidated Health Economic Evaluation Reporting Standards (CHEERS) checklist, Mendivil et al. also criticized the choice of models and the methods used to identify effectiveness data as being the least well reported [26].

It is uncertain to what extent the effects of sigmoidoscopy and specifically the differences to colonoscopy were adequately represented in models. This is also reflected in the large heterogeneity of contributing factors. Differences in modeling approaches and structural representation of CRC development, as well as the superficial consideration of localization, made it difficult to compare results. Only 1 study included alternative pathways of CRC development in its modeling [59]. In contrast, the majority reduced carcinogenesis to the adenoma-carcinoma sequence. This is particularly critical because recent analyses have found differential efficacy of sigmoidoscopy by subgroups [61]. According to this analysis, sigmoidoscopy is an effective screening measure especially for men and younger women. In women older than 60 years, the effectiveness of sigmoidoscopy in reducing mortality and incidence decreased significantly. This was attributed in part to the different incidence of distal and proximal carcinomas in men and women, which in turn is influenced by different histology and carcinogenesis. Proximal advanced neoplasms without distal lesions are more common in women than in men [62, 63]. Consequently, effectiveness of sigmoidoscopy in men and younger women as an important input parameter might be underestimated in the previous modeling approaches, leading to a systematic underestimation of its cost-effectiveness. Future models should consider the age- and sex-specific data at least in sensitivity analyses.

In addition, sigmoidoscopy is a less standardized procedure that can reach either to the descending colon or up to the left flexure [64]. In some cases, biopsies were taken during sigmoidoscopies, while in other cases this exclusively took place during colonoscopies. Often, the models left unclear what assumptions were made in this regard. There is uncertainty as to what accounts for the differences in test characteristics of sigmoidoscopy in the models. Moreover, it should be noted that, similarly to stool tests, sigmoidoscopies must be followed by a complete colonoscopy in case of abnormal findings. This is necessary because the characteristics of adenomas found in the rectum and sigmoid correlate with the likelihood of the presence of proximal neoplasia [65]. The cost of colonoscopy is assumed to be significantly variable, with implications for cost-effectiveness [28]. For example, in France, the presence of an anesthesiologist is a requirement, resulting in significantly higher colonoscopy costs in contrast to countries that do not have such a provision [49]. The overall cost structure for screening and treatment cost is affected by multiple factors, which can lead to significant international differences. Even for models from the same country, variations in costs are significant contributors to differences in cost-effectiveness.

The nature of decision-analytic models is a simplification of reality. Thus, the investigated studies often used ideal-typical assumptions, such as complete participation and adherent behavior at follow-up. Annual stool tests in particular require a high degree of reliability. Although noninvasive screening methods have higher participation than endoscopic methods [19, 66], complete participation and adherence by a large proportion of the relevant population is an idealized presumption. SAs of the models identified adherence as one of the major factors influencing cost-effectiveness. Bibliometric analysis of CRC models also supported this finding [26]. In view of this, patient behavior regarding the choice of screening method is a key determinant when considering the early detection of CRC. It is expected that the willingness to participate is higher for procedures that correspond to individual preferences. Preference elicitations for CRC screening revealed dissent on the preferred measure and differences by subgroups [67–69]. No model compared systems that offered multiple methods patients could choose between based on their age and sex. This creates a relevant research gap, as the real implications on health care systems remain vague.

The present systematic review has limitations. The restriction of the selected databases may have led to a systematic omission of studies. In addition, the review did not limit the origin of the models. The included countries differ significantly in their health care policies and in income of the population. The majority of studies reviewed were from the USA. This could have led to a bias in the results. The models set different cost-effectiveness thresholds, which were not included in the review. As various screening strategies in terms of intervals and combinations were analyzed in the models, the number of studies per comparison was low in some cases. Comparability is therefore limited. The influencing factors and drivers of cost-effectiveness could not be evaluated quantitatively due to the great heterogeneity of the studies. This also makes general clinical application of the results rather difficult.

## Conclusions

Sigmoidoscopy is a reasonable and cost-effective method for detection and prevention of CRC. Yet, in direct head-to-head comparison with other screening methods, it is often dominated. When combined with stool testing, however, it shows a significant gain in benefit at a low cost per utility gained. Further research is necessary to examine whether this combination should be promoted more and recommended in guidelines. Particularly for health care systems where multiple methods are available, sigmoidoscopy may represent a method preferred by subgroups. However, there is a lack of policy models that extend beyond a mere comparison of technologies and include preferences of the relevant population. Future decision-analytical models should incorporate such behavioral economic considerations. The analysis of subgroups will provide an essential contribution to current understanding and thus be of particular importance for decision-makers in different health care settings.

## Supporting information

S1 Checklist PRISMA checklist. (PDF)

S1 Fig Search strategy. (TIF)

S1 Table Search syntax. (PDF)

S2 Table Articles excluded in full text screening. (PDF)

S1 File Additional results. (PDF)
