# Supplementary figures and images for: Is there a place for sigmoidoscopy in colorectal cancer screening? A systematic review and critical appraisal of cost-effectiveness models
Source: PLoS One. 2023 Aug 18;18(8):e0290353. doi: 10.1371/journal.pone.0290353 (PMC10438011; doi:10.1371/journal.pone.0290353)

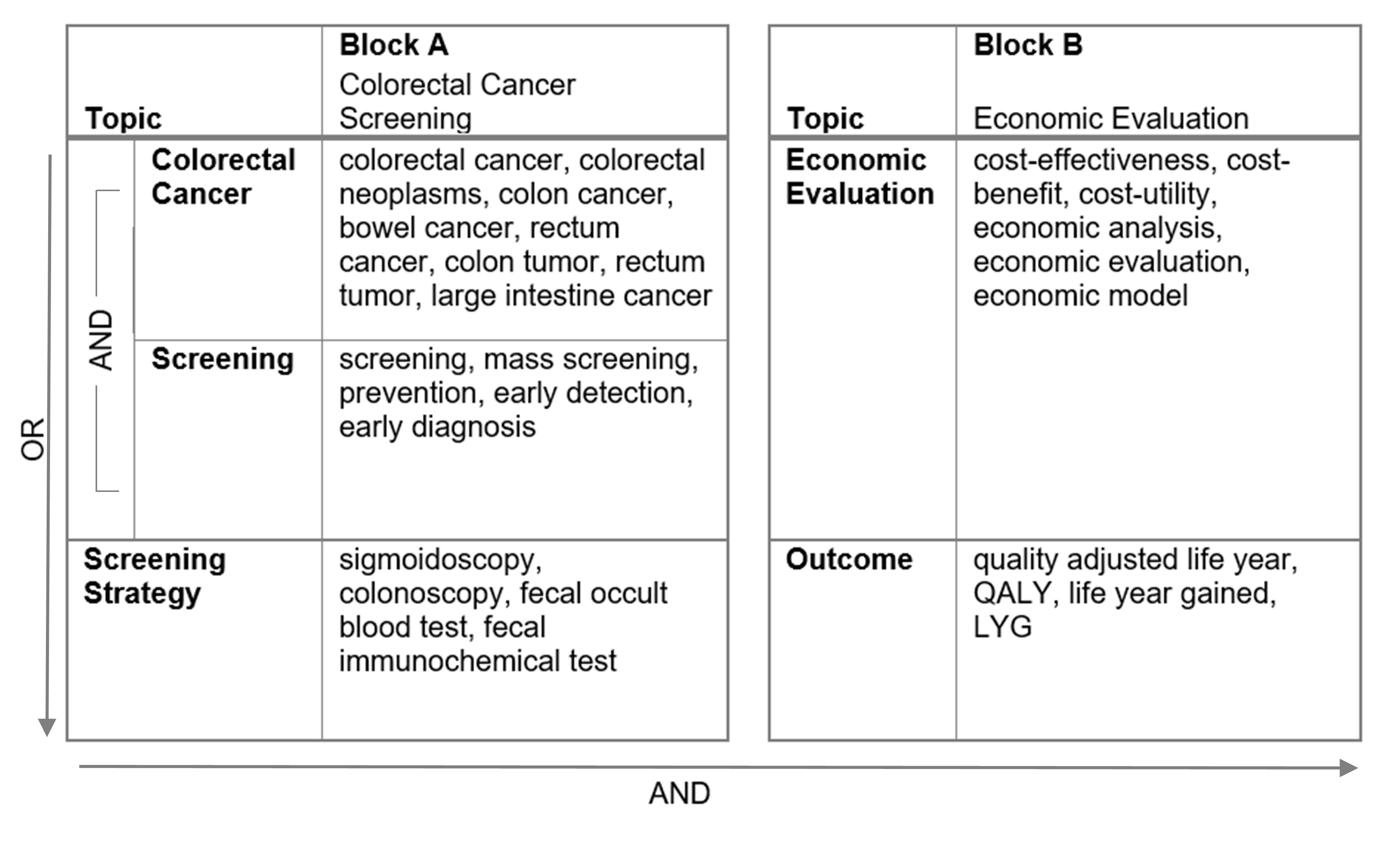

Supplement: S1 Fig — (TIF) [file pone.0290353.s002.tif]
