# Supplementary material for: Is there a place for sigmoidoscopy in colorectal cancer screening? A systematic review and critical appraisal of cost-effectiveness models
Source: PLoS One. 2023 Aug 18;18(8):e0290353. doi: 10.1371/journal.pone.0290353 (PMC10438011; doi:10.1371/journal.pone.0290353)
Supplement: S1 Table — (PDF) [file pone.0290353.s003.pdf]

Syntax of Original Search in March 2020

Embase

#	Search Query	Results
1	'colon cancer'/exp OR 'colon cancer'	280221
2	'rectum cancer'/exp OR 'rectum cancer'	214517
3	'colon tumor'/exp OR 'colon tumor'	325572
4	'rectum tumor'/exp OR 'rectum tumor'	256831
5	(colorect* OR colon* OR rect* OR bowel* OR ‘large intestine’) NEAR/3 (neoplas* OR carcinom* OR cancer* OR tumor*)	399912
6	**#1 OR #2 OR #3 OR #4 OR #5**	419321
7	'early cancer diagnosis'/exp OR 'early cancer diagnosis'	6520
8	'cancer screening'/exp OR 'cancer screening'	89933
9	screen* OR prevent*	4299302
10	early NEAR/3 detect*	127863
11	**#7 OR #8 OR #9 OR 10**	4378028
12	'colonoscopy'/exp OR 'colonoscop*'	87365
13	'sigmoidoscopy'/exp OR 'sigmoidoscop*'	14320
14	fec* OR faec*	284633
15	occult* AND blood*	22127
16	immunochemical*	22345
17	test*	5180282
18	#15 OR #16	42614
19	#14 AND #17 AND #18	8127
20	**#12 OR #13 OR #19**	98088
21	'economic evaluation'/exp OR 'economic* evaluat*'	303775
22	“economic* analys*”	10120
23	“cost* effectiv*”	246447
24	“cost* benefi*”	90371
25	„cost* utilit*“	11671
26	'economic model'/exp OR 'economic* model*'	6470
27	**#21 OR #22 OR #23 OR #24 OR #25 OR #26**	398168

28	'quality adjusted life year'/exp OR 'quality adjusted life year#'	27102
29	QALY	16216
30	'life year# gained' OR LYG	3123
31	**#28 OR #29 OR #30**	31743
32	#6 AND #10	94918
33	#20 OR #32	169688
34	#27 OR #31	402677
35	#33 AND #34	5163
36	#35 NOT [medline]/lim AND [1-1-2000]/sd AND ('article'/it OR 'article in press'/it OR 'conference abstract'/it OR 'conference paper'/it OR 'conference review'/it OR 'review'/it)	1605

Medline (via Ovid)

Ovid MEDLINE(R) and Epub Ahead of Print, In-Process & Other Non-Indexed Citations, Daily and Versions(R)

#	Search Query	Results
1	exp Colorectal Neoplasms/	197528
2	(colon* adj3 (neoplas* or carcinom* or cancer* or tumor*)).mp.	114811
3	(colorect* adj3 (neoplas* or carcinom* or cancer* or tumor*)).mp.	138020
4	(rect* adj3 (neoplas* or carcinom* or cancer* or tumor*)).mp.	52716
5	(bowel* adj3 (neoplas* or carcinom* or cancer* or tumor*)).mp. [	5430
6	("large intestin*" adj3 (neoplas* or carcinom* or cancer* or tumor*)).mp.	1337
7	**1 or 2 or 3 or 4 or 5 or 6**	267976
8	exp Mass Screening/	125865
9	exp "Early Detection of Cancer"/	24160
10	(screen* or prevent*).mp.	3009464
11	(early adj3 detect*).mp.	106569
12	**8 or 9 or 10 or 11**	3075805
13	exp Colonoscopy/	29587
14	(((fec* or faec*) and ((occult* and blood*) or immunochemical*)) adj2 test*).mp.	4540
15	(colonoscop* or sigmoidosc*).mp.	45237
16	**13 or 14 or 15**	47048

17	exp "Costs and Cost Analysis"/	233508
18	exp Models, Economic/	14775
19	economic* evaluat*.mp.	12330
20	economic* analys*.mp.	6791
21	cost* effectiv*.mp.	132648
22	cost* benef*.mp.	87089
23	cost* utilit*.mp.	4793
24	(economic* adj3 model*).mp.	13769
25	**17 or 18 or 19 or 20 or 21 or 22 or 23 or 24**	339181
26	exp Quality-Adjusted Life Years/	11886
27	("quality adjusted life year$1" or QALY).mp.	18514
28	("life year$1 gained" or LYG).mp.	2756
29	**26 or 27 or 28**	19646
30	7 and 12	52490
31	16 or 30	87413
32	25 or 29	342127
33	31 and 32	2685
34	limit 33 to (yr="2000 -Current" and (english or german))	2061
35	limit 33 to (address or autobiography or bibliography or biography or comment or dictionary or directory or editorial or "expression of concern" or festschrift or historical article or interactive tutorial or interview or lecture or legal case or legislation or letter or news or newspaper article or patient education handout or personal narrative or portrait or video-audio media or webcast)	217
36	34 not 35	1897

Web of Science Core Collection

#	Search Query	Results
1	ts=(colon* near/3 (neoplas* or carcinom* or cancer* or tumor*))<br>Indexes=SCI-EXPANDED, SSCI Timespan=All years	116,410
2	ts=(colorect* near/3 (neoplas* or carcinom* or cancer* or tumor*))<br>Indexes=SCI-EXPANDED, SSCI Timespan=All years	193,210
3	ts=(rect* near/3 (neoplas* or carcinom* or cancer* or tumor*))<br>Indexes=SCI-EXPANDED, SSCI Timespan=All years	39,231
4	ts=(bowel* near/3 (neoplas* or carcinom* or cancer* or tumor*))<br>Indexes=SCI-EXPANDED, SSCI Timespan=All years	7,848

5	ts=("large intestin*" near/3 (neoplas* or carcinom* or cancer* or tumor*))<br>Indexes=SCI-EXPANDED, SSCI Timespan=All years	654
6	**#5 OR #4 OR #3 OR #2 OR #1**<br>Indexes=SCI-EXPANDED, SSCI Timespan=All years	291,681
7	ts=(screen* OR prevent*)<br>Indexes=SCI-EXPANDED, SSCI Timespan=All years	2,244,403
8	ts=(early near/3 detect*)<br>Indexes=SCI-EXPANDED, SSCI Timespan=All years	87,899
9	**#8 OR #7**<br>*Indexes=SCI-EXPANDED, SSCI Timespan=All years*	2,306,488
10	ts=(colonoscop*)<br>Indexes=SCI-EXPANDED, SSCI Timespan=All years	34,300
11	ts=(sigmoidosc*)<br>Indexes=SCI-EXPANDED, SSCI Timespan=All years	5,020
12	ts=(fec* OR faec*)<br>Indexes=SCI-EXPANDED, SSCI Timespan=All years	214,969
13	ts=(occult* AND blood*)<br>Indexes=SCI-EXPANDED, SSCI Timespan=All years	9,017
14	ts=(immunochemical*)<br>Indexes=SCI-EXPANDED, SSCI Timespan=All years	17,186
15	ts=(test*)<br>Indexes=SCI-EXPANDED, SSCI Timespan=All years	4,458,007
16	#14 OR #13<br>Indexes=SCI-EXPANDED, SSCI Timespan=All years	25,135
17	#16 AND #15 AND #12<br>Indexes=SCI-EXPANDED, SSCI Timespan=All years	5,007
18	**#17 OR #11 OR #10**<br>*Indexes=SCI-EXPANDED, SSCI Timespan=All years*	39,117
19	ts=("economic* evaluat*" OR "cost* effectiv*" OR "cost* benef*" OR "cost* utilit*" OR "economic* analys*")<br>*Indexes=SCI-EXPANDED, SSCI Timespan=All years*	255,246
20	ts=(economic* Near/3 model*)<br>*Indexes=SCI-EXPANDED, SSCI Timespan=All years*	24,505
21	**#20 OR #19**<br>*Indexes=SCI-EXPANDED, SSCI Timespan=All years*	275,423
22	ts=("quality adjusted life year*" OR QALY)<br>*Indexes=SCI-EXPANDED, SSCI Timespan=All years*	12,163
23	ts=("life year* gained" OR LYG)<br>*Indexes=SCI-EXPANDED, SSCI Timespan=All years*	2,362
24	**#23 OR #22**<br>*Indexes=SCI-EXPANDED, SSCI Timespan=All years*	13,235
25	*#9 AND #6*<br>*Indexes=SCI-EXPANDED, SSCI Timespan=All years*	45,828
26	#25 OR #18<br>Indexes=SCI-EXPANDED, SSCI Timespan=All years	74,439
27	#24 OR #21<br>Indexes=SCI-EXPANDED, SSCI Timespan=All years	277,073
28	*#27 AND #26*	2,304
29	(#28) AND LANGUAGE: (English OR German) AND DOCUMENT TYPES: (Article OR Abstract of Published Item OR Data Paper OR Proceedings Paper OR Record Review OR Review)<br>Indexes=SCI-EXPANDED, SSCI Timespan=2000-2020	1,782

EconLit

#	Search Query	Results
S1	colon* N3 (neoplas* OR carcinom* OR cancer* OR tumor*)	33
S2	colorect* N3 (neoplas* OR carcinom* OR cancer* OR tumor*)	100
S3	rect* N3 (neoplas* OR carcinom* OR cancer* OR tumor*)	4
S4	bowel* N3 (neoplas* OR carcinom* OR cancer* OR tumor*)	6
S5	intestin* N3 (neoplas* OR carcinom* OR cancer* OR tumor*)	1
S6	S1 OR S2 OR S3 OR S4 OR S5	128
S7	screen* OR prevent* OR (early N3 detect*)	20610
S8	colonoscop* OR sigmoidosc*	29
S9	(fec* OR faec*) AND ((occult* AND blood*) OR immunochemical*) AND test*	12
S10	S8 OR S9	34
S11	economic* evaluat*	5145
S12	economic* analys*	88656
S13	cost* effectiv*	6509
S14	cost* benef*	24365
S15	cost* utilit*	1196
S16	economic* N3 model*	29004
S17	S11 OR S12 OR S13 OR S14 OR S15 OR S16	145970
S18	"quality adjusted life year*" OR QALY	532
S19	"life year* gained" OR LYG	86
S20	S18 OR S19	579
S21	S6 AND S7	73
S22	S10 OR S21	88
S23	S17 OR S20	146148
S24	S22 AND S23<br><br>Limiters - Published Date: 20000101-20201231<br><br>Expanders - Apply equivalent subjects<br><br>Search modes - Boolean/Phrase	28

Cost-Effectiveness Analysis (CEA) Registry (Tufts Medical Center)

#	Search Query	Results
1	Colorectal cancer screening	23
2	Bowel Cancer Screening	4
3	Early detection colorectal cancer	0
4	Colon cancer screening	2

British National Health Service Economic Evaluation Database (NHS EED)

#	Search Query	Results
1	((colorect* OR colon* OR rect* OR bowel*) AND (neoplas* OR carcinom* OR cancer* OR tumor*)) AND (screen* OR prevent*)	284
