# Supplementary material for: Is there a place for sigmoidoscopy in colorectal cancer screening? A systematic review and critical appraisal of cost-effectiveness models
Source: PLoS One. 2023 Aug 18;18(8):e0290353. doi: 10.1371/journal.pone.0290353 (PMC10438011; doi:10.1371/journal.pone.0290353)
Supplement: S2 Table — (PDF) [file pone.0290353.s004.pdf]

Articles excluded in full text screening

Authors	Year	Title	Journal	DOI	Reason for exclusion
Allameh, Zahra; Davari, Majid; Emami, Mohammad Hasan	2011	Cost-Effectiveness Analysis of Colorectal Cancer Screening Methods in Iran	Arch Iran Med (Archives of Iranian medicine)		4
Anand, V.; Gwinnett, J.; Phillips, A.	2012	Survival benefit of increasing colorectal cancer screening uptake in Wolverhampton, UK: An exploratory study	Public Health (Public health)	10.1016/j.puhe.2012.05.022	6
Areia M.; Fuccio L.; Hassan C.; Dekker E.; Dias-Pereira A.; Dinis-Ribeiro M.	2018	Cost-utility analysis of colonoscopy or faecal immunochemical test for colorectal cancer screening	United European Gastroenterol. j. (United European gastroenterology journal)	10.1177/2050640618792817	6
Areia M.; Fuccio L.; Hassan C.; Dekker E.; Dias-Pereira A.; Dinis-Ribeiro M.	2019	Cost-utility analysis of colonoscopy or faecal immunochemical test for population-based organised colorectal cancer screening	United European Gastroenterol. j. (United European gastroenterology journal)	10.1177/2050640618803196	2
Aronsson M.; Carlsson P.; Ekbom A.; Hultcrantz R.; Forsberg A.	2016	Health effects and costs due to postcolonoscopy colorectal cancer	United European Gastroenterol. j. (United European gastroenterology journal)	10.1177/2050640616663688	6
Aronsson, M.; Carlsson, P.; Levin, L.-A.; Hager, J.; Hultcrantz, R.	2017	Cost-effectiveness of high-sensitivity faecal immunochemical test and colonoscopy screening for colorectal cancer	Br J Surg (The British journal of surgery)	10.1002/bjs.10536	2
Arrospide, Arantzazu; Idigoras, Isabel; Mar, Javier; Koning, Harry de; van der Meulen, Miriam; Soto-Gordoa, Myriam; Miguel Martinez-Llorente, Jose; Portillo, Isabel; Arana-Arri, Eunate; Ibarrondo, Oliver; Lansdorp-Vogelaar, Iris	2018	Cost-effectiveness and budget impact analyses of a colorectal cancer screening programme in a high adenoma prevalence scenario using MISCAN-Colon microsimulation model	BMC Cancer (BMC cancer)	10.1186/s12885-018-4362-1	2

Asaria M.; Griffin S.; Cookson R.; Tappenden P.; Whyte S.	2013	A framework for the cost-equality analysis of health care programmes	Value Health (Value in health : the journal of the International Society for Pharmacoeconomics and Outcomes Research)	10.1016/j.jval.2013.08.591		6
Asaria, Miqdad; Griffin, Susan; Cookson, Richard	2016	Distributional Cost-Effectiveness Analysis: A Tutorial	Med Decis Making (Medical decision making : an international journal of the Society for Medical Decision Making)	10.1177/0272989X15583266		2
Austin, Gregory L.; Fennimore, Blair; Ahnen, Dennis J.	2013	Can Colonoscopy Remain Cost-Effective for Colorectal Cancer Screening? The Impact of Practice Patterns and the Will Rogers Phenomenon on Costs	Am J Gastroenterol (The American journal of gastroenterology)	10.1038/ajg.2012.195		2
Azad, Nilofer S.; Leeds, Ira L.; Wanjau, Waruguru; Shin, Eun J.; Padula, William V.	2020	Cost-utility of colorectal cancer screening at 40years old for average-risk patients	Prev Med (Preventive medicine)	10.1016/j.ypmed.2020.106003		3
Barichello, Scott; Deng, Lu; Ismond, Kathleen P.; Loomes, Dustin E.; Kirwin, Erin M.; Wang, Haili; Chang, David; Svenson, Lawrence W.; Nguyen Xuan Thanh	2019	Comparative effectiveness and cost-effectiveness analysis of a urine metabolomics test vs. alternative colorectal cancer screening strategies	Int J Colorectal Dis (International journal of colorectal disease)	10.1007/s00384-019-03419-7		2
Barouni, Mohsen; Ghaderi, Hosien; Shahmoradi, Mohammad Kazem	2013	The Economic Evaluation of Screening for Colorectal Cancer: Case of Iran	Clin Lab (Clinical laboratory)	10.7754/Clin.Lab.2012.120812		2
Barouni, Mohsen; Larizadeh, Mohammad Hassan; Sabermahani, Asma; Ghaderi, Hossien	2012	Markov's Modeling for Screening Strategies for Colorectal Cancer	Asian Pac J Cancer Prev (Asian Pacific journal of cancer prevention : APJCP)	10.7314/APJCP.2012.13.10.5125		2
Barzi A.; Directo M.; Dasu S.; Lenz H.-J.; Sadeghi S.	2013	Saving lives and costs through screening (Sc) for colorectal cancer (CRC): Implications for limited-resource healthcare systems (LRHS)	J Clin Oncol (Journal of clinical oncology : official journal of the American Society of Clinical Oncology)			6

Barzi A.; Lenz H.-J.; Quinn D.I.; Sadeghi S.	2015	Colonoscopy versus less invasive approaches for colorectal cancer (CRC) screening (Scr): A strategic perspective	J Clin Oncol (Journal of clinical oncology : official journal of the American Society of Clinical Oncology)			6
Barzi A.; Quinn D.I.; Idos G.; Lenz H.-J.; Sadeghi S.	2016	The opportunity cost (OC) of low adherence with screening (scr) recommendations for colorectal cancer (CRC)	Journal of Clinical Oncology			6
Barzi A.; Sadeghi S.	2014	Value proposition for colorectal cancer (CRC) screening (SC) in underinsured populations (UP)	J Clin Oncol (Journal of clinical oncology : official journal of the American Society of Clinical Oncology)			6
Barzi A.; Siegel R.; Fedewa S.; Lenz H.J.; Quinn D.; Jemal A.; Sadeghi S.	2017	Comparative effectiveness of initiating colorectal cancer (CRC) screening (scr) at age 45	European Journal of Cancer			6
Berger, Barry M.; Schroy, Paul C. 3rd; Dinh, Tuan A.	2016	Screening for Colorectal Cancer Using a Multitarget Stool DNA Test: Modeling the Effect of the Intertest Interval on Clinical Effectiveness	Clin Colorectal Cancer (Clinical colorectal cancer)	10.1016/j.clcc.2015.12.003		2
Bhattacharya R.; Shah J.; Sansgiry S.	2010	Comparison of advanced noninvasive techniques to screen colorectal cancer: Fecal immunochemical test vs. fecal DNA; A cos-effectiveness study	Value Health (Value in health : the journal of the International Society for Pharmacoeconomics and Outcomes Research)			6
Biltaji E.; Au T.; Walker B.; Ose J.; Ulrich C.M.; Stenehjem D.; Brixner D.	2017	Cost threshold analysis of genotype-guided aspirin use for colorectal cancer prevention	Value in Health			6

Biltaji E.; Au T.H.; Walker B.; Ose J.; Ulrich C.M.; Stenehjem D.D.; Brixner D.I.	2017	Cost-effectiveness of genotype-guided aspirin use for colorectal cancer prevention	J Clin Oncol (Journal of clinical oncology : official journal of the American Society of Clinical Oncology)			6
Blaylock B.; Hay J.; Zarchy T.	2012	The cost-effectiveness of diagnostic sigmoidoscopy before colonoscopy in 40 to 49-year-old symptomatic patients	Value Health (Value in health : the journal of the International Society for Pharmacoeconomics and Outcomes Research)	10.1016/j.jval.2012.03.369		6
Boltin D.; Niv Y.	2012	Is there a place for screening flexible sigmoidoscopy?	Curr. colorectal cancer rep. (Current colorectal cancer reports)	10.1007/s11888-011-0108-z		5
Bradley, Cathy J.; Lansdorp-Vogelaar, Iris; Yabroff, Robin; Dahman, Bassam; Mariotto, Angela; Feuer, Eric J.; Brown, Martin L.	2011	Productivity Savings from Colorectal Cancer Prevention and Control Strategies	Am J Prev Med (American journal of preventive medicine)	10.1016/j.amepre.2011.04.008		3
Bronzwaer, Maxime E. S.; Greuter, Marjolein J. E.; Bleijenberg, Arne G. C.; IJspeert, Joep E. G.; Dekker, Evelien; Coupe, Veerle M. H.	2018	Impact of differences in adenoma and proximal serrated polyp detection rate on the long-term effectiveness of FIT-based colorectal cancer screening	BMC Cancer (BMC cancer)	10.1186/s12885-018-4375-9		2
Burnett-Hartman A.; Newcomb P.; Veenstra D.	2013	The impact of colonoscopy screening guidelines that incorporate precursors in the serrated pathway: A cost-effectiveness analysis	Cancer Epidemiol Biomarkers Prev (Cancer epidemiology, biomarkers & prevention : a publication of the American Association for Cancer Research, cosponsored by the American Society of Preventive Oncology)	10.1158/1055-9965.EPI-13-0081		6
Byun, Ju-Young; Yoon, Seok-Jun; Oh, In-Hwan; Kim, Young Ae; Seo, Hye-Young; Lee, Yo-Han	2014	Economic burden of colorectal cancer in Korea	J Prev Med Pub Health (Journal of preventive medicine and public health = Yebang Uihakhoe chi)	10.3961/jpmph.2014.47.2.84		2

Campbell, Leslie Anne; Blake, John T.; Kephart, George; Grunfeld, Eva; MacIntosh, Donald	2017	Understanding the Effects of Competition for Constrained Colonoscopy Services with the Introduction of Population-level Colorectal Cancer Screening: A Discrete Event Simulation Model	Med Decis Making (Medical decision making : an international journal of the Society for Medical Decision Making)	10.1177/0272989X16670638		5
Castells A.; Serradesanferm A.; Pellisé M.; Augé J.M.; Polbach S.; Grau J.; Trilla A.	2011	Early detection of colorectal cancer: An update	Revisiones en Cancer			7
Cenin D.; Naber S.; De Weerdt A.; Jenkins M.; Preen D.; Ee H.; O'Leary P.; Lansdorp-Vogelaar I.	2018	Screening for colorectal cancer based on polygenic risk and family history	United European Gastroenterol. j. (United European gastroenterology journal)	10.1177/2050640618792817		6
Cenin, Dayna R.; Naber, Steffie K.; Weerdt, Anne C. de; Jenkins, Mark A.; Preen, David B.; Ee, Hooi C.; O'Leary, Peter C.; Lansdorp-Vogelaar, Iris	2020	Cost-Effectiveness of Personalized Screening for Colorectal Cancer Based on Polygenic Risk and Family History	Cancer Epidemiol Biomarkers Prev (Cancer epidemiology, biomarkers & prevention : a publication of the American Association for Cancer Research, cosponsored by the American Society of Preventive Oncology)	10.1158/1055-9965.EPI-18-1123		2
Chauvin, Pauline; Josselin, Jean-Michel; Heresbach, Denis	2012	Incremental net benefit and acceptability of alternative health policies: a case study of mass screening for colorectal cancer	Eur J Health Econ (The European journal of health economics : HEPAC : health economics in prevention and care)	10.1007/s10198-011-0300-8		2
Chauvin, Pauline; Josselin, Jean-Michel; Heresbach, Denis	2014	The influence of waiting times on cost-effectiveness: a case study of colorectal cancer mass screening	Eur J Health Econ (The European journal of health economics : HEPAC : health economics in prevention and care)	10.1007/s10198-013-0525-9		2
Chen K.-Y.	2010	Colon-rectal cancer screening-early detection	International Journal of Urology	10.1111/j.1442-2042.2010.02646.x		6

Coldman A.; Flanagan W.; Nadeau C.; Wolfson M.; Fitzgerald N.; Memon S.; Gauvreau C.; Miller A.; Earle C.	2017	Projected effect of fecal immunochemical test threshold for colorectal cancer screening on outcomes and costs for Canada using the OncoSim microsimulation model	Journal of Cancer Policy	10.1016/j.jcpo.2017.07.004	2
Coldman A.J.; Phillips N.; Brisson J.; Flanagan W.; Wolfson M.; Nadeau C.; Fitzgerald N.; Miller A.B.	2015	Using the cancer risk management model to evaluate colorectal cancer screening options for Canada	Curr. oncol. (Current oncology (Toronto, Ont.))	10.3747/co.22.2013	4
Comas, Merce; Mendivil, Joan; Andreu, Montserrat; Hernandez, Cristina; Castells, Xavier	2016	Long-Term Prediction of the Demand of Colonoscopies Generated by a Population-Based Colorectal Cancer Screening Program	PLoS ONE (PloS one)	10.1371/journal.pone.0164666	2
D'Andrea, Elvira; Ahnen, Dennis J.; Sussman, Daniel A.; Najafzadeh, Mehdi	2020	Quantifying the impact of adherence to screening strategies on colorectal cancer incidence and mortality	Cancer Med (Cancer medicine)	10.1002/cam4.2735	5
Davis, Melinda M.; Nambiar, Siddhartha; Mayorga, Maria E.; Sullivan, Eliana; Hicklin, Karen; O'Leary, Meghan C.; Dillon, Kristen; Lich, Kristen Hassmiller; Gu, Yifan; Lind, Bonnie K.; Wheeler, Stephanie B.	2019	Mailed FIT (fecal immunochemical test), navigation or patient reminders? Using microsimulation to inform selection of interventions to increase colorectal cancer screening in Medicaid enrollees	Prev Med (Preventive medicine)	10.1016/j.ypmed.2019.105836	2
den Hoed C.M.; Isendoorn K.; Klinkhamer W.; Gupta A.M.; Kuipers E.J.	2011	The valuation of the increase in quality of life and health-adjusted life expectancy as a result of colorectal cancer screening in future decades; a population-based study	Gastroenterology (Gastroenterology)	10.1016/S0016-5085(11)61661-2	6

Di Bidino, Rossella; Cicchetti, Americo; Corio, Mirella; Polisena, Julie; Oradei, Marco; Marchetti, Marco	2010	Impact of technology overlapping: A case study on colorectal cancer screening	Technol Health Care (Technology and health care : official journal of the European Society for Engineering and Medicine)	10.3233/THC-2010-0594		3
Dillon, Mary; Flander, Louisa; Buchanan, Daniel D.; Macrae, Finlay A.; Emery, Jon D.; Winship, Ingrid M.; Boussioutas, Alex; Giles, Graham G.; Hopper, John L.; Jenkins, Mark A.; Ait Ouakrim, Driss	2018	Family history-based colorectal cancer screening in Australia: A modelling study of the costs, benefits, and harms of different participation scenarios	PLoS Med (PLoS medicine)	10.1371/journal.pmed.1002630		2
Ding, Hanyue; Mao, Ayan; Wen, Lian; Dong, Pei; Yang, Yujie; Cheng, Xuan; Qiu, Wuqi	2019	Cost-utility analysis of screening for colorectal precancerous lesions and cancer in Beijing: A case-control study	J Cancer Res Ther (Journal of cancer research and therapeutics)	10.4103/jcrt.JCRT_194_19		2
Dinh T.; Alperin P.; Caldwell C.; Levin T.R.	2010	Cost effectiveness analysis of hybrid screening strategies for colorectal cancer in managed care setting	Gastroenterology (Gastroenterology)			6
Dou G.; Li X.; Ying X.	2018	COST-EFFECTIVENESS SIMULATION AND ANALYSIS OF COLORECTAL CANCER SCREENING IN SHANGHAI POPULATION, CHINA: COMPARISON BETWEEN URBAN AND RURAL RESIDENTS	Value Health (Value in health : the journal of the International Society for Pharmacoeconomics and Outcomes Research)	10.1016/j.jval.2018.09.230		6
Duddalwar V.; Quinn D.I.; Lenz H.-J.; Barzl A.; Sadeghi S.	2016	Comparative effectiveness of CT colonography (CT-C) as a screening (Scr) tool for colorectal cancer (CRC)	Journal of Clinical Oncology			6
Dunkle A.J.; Blackwell M.; Jaros R.; Kohn S.; Stucke A.; Volkov F.; Wang A.; Niclasen B.	2017	Colorectal cancer screening in greenland-an economic model	Annals of Global Health			6

Erenay, Fatih Safa; Alagoz, Oguzhan; Said, Adnan	2014	Optimizing Colonoscopy Screening for Colorectal Cancer Prevention and Surveillance	Manufacturing and Service Operations Management	10.1287/msom.2014.0484		2
Evans W.K.; Wolfson M.; Flanagan W.; Oderkirk J.; Goffin J.R.; Shin J.; Lockwood G.	2011	Canadian cancer risk management model: A new health policy tool useful in policy decisions related to lung cancer	J Clin Oncol (Journal of clinical oncology : official journal of the American Society of Clinical Oncology)			6
Fitch, Kathryn; Pyenson, Bruce; Blumen, Helen; Weisman, Thomas; Small, Art	2015	The Value of Colonoscopic Colorectal Cancer Screening of Adults Aged 50 to 64 Years	Am J Manag Care (The American journal of managed care)			2
Fitzgerald N.; Gauvreau C.; Memon S.; Hussain S.; Coldman A.; Popadiuk C.; Evans W.; Wolfson M.; Flanagan W.; Nadeau C.; Asakawa K.; Garner R.; Miller A.	2018	The oncosim cancer simulation platform: A tool to project the population effects of cancer control interventions in Canada	J. glob. oncol. (Journal of global oncology)	10.1200/jgo.18.20300		6
Folse H.J.; Dinh T.	2014	Stratified cost-effectiveness analysis to guide genetic screening for cancer risk	Value Health (Value in health : the journal of the International Society for Pharmacoeconomics and Outcomes Research)	10.1016/j.jval.2014.08.1875		6
Gauvreau C.L.; Fitzgerald N.R.; Memon S.; Flanagan W.M.; Nadeau C.; Asakawa K.; Garner R.; Miller A.B.; Evans W.K.; Popadiuk C.M.; Wolfson M.; Coldman A.J.	2017	The OncoSim model: Development and use for better decision-making in Canadian cancer control	Curr. oncol. (Current oncology (Toronto, Ont.))	10.3747/co.24.3850		5
Gerson L.B.; Jackson C.S.	2012	Cost-effectiveness of enteroscopy versus CTC for incomplete colonoscopy	Gastrointest Endosc (Gastrointestinal endoscopy)	10.1016/j.gie.2012.03.1098		6
Ginsberg G.M.; Lim S.S.; Lauer J.A.; Johns B.P.; Sepulveda C.R.	2010	Prevention, screening and treatment of colorectal cancer: A global and regional generalized cost effectiveness analysis	Cost Eff Resour Alloc (Cost effectiveness and resource allocation : C/E)	10.1186/1478-7547-8-2		3

Ginsberg, Gary M.; Lauer, Jeremy A.; Zelle, Sten; Baeten, Steef; Baltussen, Rob	2012	Cost effectiveness of strategies to combat breast, cervical, and colorectal cancer in sub-Saharan Africa and South East Asia: mathematical modelling study	BMJ-BRITISH MEDICAL JOURNAL	10.1136/bmj.e614		3
Glover M.; Buxton M.; Guthrie S.; Hanney S.; Pollitt A.; Grant J.	2014	Estimating the returns to UK publicly funded cancer-related research in terms of the net value of improved health outcomes	BMC Med (BMC medicine)	10.1186/1741-7015-12-99		5
Goede L.; Knudsen A.; Van Ballegooijen M.; Kuntz K.; Lansdorp-Vogelaar I.; Tangka F.; Howard D.; Zauber A.; Seeff L.	2010	Cost-savings to medicare from increased colorectal cancer screening in the pre-medicare population 2010 ACG international award	Am J Gastroenterol (The American journal of gastroenterology)	10.1038/ajg.2010.320-8		6
Goede, S. Lucas; Rabeneck, Linda; van Ballegooijen, Marjolein; Zauber, Ann G.; Paszat, Lawrence F.; Hoch, Jeffrey S.; Yong, Jean H. E.; Kroep, Sonja; Tinmouth, Jill; Lansdorp-Vogelaar, Iris	2017	Harms, benefits and costs of fecal immunochemical testing versus guaiac fecal occult blood testing for colorectal cancer screening	PLoS ONE (PloS one)	10.1371/journal.pone.0172864		2
Goede, S. Lucas; van Roon, Aafke H C; Reijerink, Jacqueline C I Y; van Vuuren, Anneke J.; Lansdorp-Vogelaar, Iris; Habbema, J. Dik F.; Kuipers, Ernst J.; van Leerdam, Monique E.; van Ballegooijen, Marjolein	2013	Cost-effectiveness of one versus two sample faecal immunochemical testing for colorectal cancer screening	Gut (Gut)	10.1136/gutjnl-2011-301917		2
Goede, Simon L.; Kuntz, Karen M.; van Ballegooijen, Marjolein; Knudsen, Amy B.; Lansdorp-Vogelaar, Iris; Tangka, Florence K.; Howard, David H.; Chin, Joseph; Zauber, Ann G.; Seeff, Laura C.	2015	Cost-Savings to Medicare From Pre-Medicare Colorectal Cancer Screening	Med Care (Medical care)	10.1097/MLR.0000000000000380		5

Gomes, Manuel; Aldridge, Robert W.; Wylie, Peter; Bell, James; Epstein, Owen	2013	Cost-effectiveness analysis of 3-D computerized tomography colonography versus optical colonoscopy for imaging symptomatic gastroenterology patients	Appl Health Econ Health Policy (Applied health economics and health policy)	10.1007/s40258-013-0019-z		2
Good, Norm M.; Suresh, Krithika; Young, Graeme P.; Lockett, Trevor J.; Macrae, Finlay A.; Taylor, Jeremy M. G.	2015	A prediction model for colon cancer surveillance data	STATISTICS IN MEDICINE	10.1002/sim.6500		5
Gopalappa, Chaitra; Guo, Jiachen; Meckoni, Prashant; Munkhbat, Buyannemekh; Pretorius, Carel; Lauer, Jeremy; Ilbawi, Andre; Bertram, Melanie	2018	A Two-Step Markov Processes Approach for Parameterization of Cancer State-Transition Models for Low- and Middle-Income Countries	Med Decis Making (Medical decision making : an international journal of the Society for Medical Decision Making)	10.1177/0272989X18759482		5
Gregorich M.G.; Urach C.; Breitenecker F.	2016	Cost-effectiveness analysis of colorectal cancer screening in Austria and assessment of the impact of cancer-causing risk factors	Value in Health			6
Greuter M.; Demirel E.; Berkhof J.; Fijneman R.; Stoker J.; Meijer G.; Coupé V.	2014	The potential of MR colonography as a screening tool for colorectal cancer: A cost-effectiveness analysis	United European Gastroenterol. j. (United European gastroenterology journal)	10.1177/2050640614548980		6
Greuter M.J.; Demirel E.; Berkhof J.; Fijneman R.J.; Stoker J.; Meijer G.A.; Coupé V.M.	2014	The potential of (targ eted) MR colonography as a screening tool for colorectal cancer: A cost-effectiveness analysis	Value Health (Value in health : the journal of the International Society for Pharmacoeconomics and Outcomes Research)	10.1016/j.jval.2014.08.2260		6
Greuter M.J.E.; Dekker E.; Meijer G.A.; Coupé V.M.H.	2016	The added benefit of surveillance in colorectal cancer screening	United European Gastroenterol. j. (United European gastroenterology journal)	10.1177/2050640616663688		6

Greuter, Marjolein J. E.; Berkhof, Johannes; Fijneman, Remond J. A.; Demirel, Erhan; Lew, Jie-Bin; Meijer, Gerrit A.; Stoker, Jaap; Coupe, Veerle M. H.	2016	The potential of imaging techniques as a screening tool for colorectal cancer: a cost-effectiveness analysis	Br J Radiol (The British journal of radiology)	10.1259/bjr.20150910		2
Greuter, Marjolein J. E.; Klerk, Clasine M. de; Meijer, Gerrit A.; Dekker, Evelien; Coupe, Veerle M. H.	2017	Screening for Colorectal Cancer With Fecal Immunochemical Testing With and Without Postpolypectomy Surveillance Colonoscopy A Cost-Effectiveness Analysis	Ann Intern Med (Annals of internal medicine)	10.7326/M16-2891		2
Hashimoto Y.; Igarashi A.; Miyake M.; Iinuma G.; Fukuda T.; Tsutani K.	2014	Cost-effectiveness analysis of CT colonography for colorectal cancer screening program to working age in Japan	Value Health Reg Issues (Value in health regional issues)	10.1016/j.vhri.2014.04.009		2
Hashimoto Y.; Igarashi A.; Miyake M.; Iinuma G.; Fukuda T.; Tsutani K.	2012	Economic evaluation of CT colonography (CTC) introduction into the colorectal cancer screening program in Japan	Value Health (Value in health : the journal of the International Society for Pharmacoeconomics and Outcomes Research)	10.1016/j.jval.2012.08.315		6
Hassan C.; Gralnek I.	2014	The cost-effectiveness of ''full spectrum endoscopy (FUSE)'' colonoscopy for colorectal cancer screening	United European Gastroenterol. j. (United European gastroenterology journal)	10.1177/2050640614548980		6
Hassan C.; Gralnek I.M.	2014	“Full spectrum endoscopy (Fuse)” colonoscopy is cost effective for colorectal cancer screening and surveillance	Gastrointest Endosc (Gastrointestinal endoscopy)	10.1016/j.gie.2014.02.914		6
Haug, Ulrike; Knudsen, Amy B.; Kuntz, Karen M.	2012	How should individuals with a false-positive fecal occult blood test for colorectal cancer be managed? A decision analysis	Int J Cancer (International journal of cancer)	10.1002/ijc.27463		2

Haug, Ulrike; Knudsen, Amy B.; Lansdorp-Vogelaar, Iris; Kuntz, Karen M.	2015	Development of new non-invasive tests for colorectal cancer screening: The relevance of information on adenoma detection	Int J Cancer (International journal of cancer)	10.1002/ijc.29343	2
Heresbach, Denis; Chauvin, Pauline; Grolier, Jacques; Josselin, Jean-Michel	2010	Cost-effectiveness of colorectal cancer screening with computed tomography colonography or fecal blood tests	Eur J Gastroenterol Hepatol (European journal of gastroenterology & hepatology)	10.1097/MEG.0b013e32833eaa71	2
Huang Q.; Ye D.; Jiang X.; Li Q.; Yao K.; Wang J.; Jin M.; Chen K.	2017	Cost-effectiveness analysis on colorectal cancer screening program	Chinese Journal of Endemiology	10.3760/cma.j.issn.0254-6450.2017.01.012	7
Huang, Weidong; Liu, Guoxiang; Zhang, Xin; Fu, Wenqi; Zheng, Shu; Wu, Qunhong; Liu, Chaojie; Liu, Yang; Cai, Shanrong; Huang, Yanqin	2014	Cost-Effectiveness of Colorectal Cancer Screening Protocols in Urban Chinese Populations	PLoS ONE (PloS one)	10.1371/journal.pone.0109150	2
Jahn, Beate; Sroczynski, Gaby; Bundo, Marvin; Muehlberger, Nikolai; Puntscher, Sibylle; Todorovic, Jovan; Rochau, Ursula; Oberaigner, Willi; Koffijberg, Hendrik; Fischer, Timo; Schiller-Fruehwirth, Irmgard; Oefner, Dietmar; Renner, Friedrich; Jonas, Michael; Hackl, Monika; Ferlitsch, Monika; Siebert, Uwe; Austrian Colorectal Canc Screening	2019	Effectiveness, benefit harm and cost effectiveness of colorectal cancer screening in Austria	BMC Gastroenterol (BMC gastroenterology)	10.1186/s12876-019-1121-y	2
Jalali A.; Nelson R.; Nirula R.	2019	Cost-effectiveness of current and potential serum based colorectal screening strategies: Can a serum based test do better	Cancer Res (Cancer research)	10.1158/1538-7445.SABCS18-3352	6
Joseph, Grace N.; Heidarnejad, Farid; Sherer, Eric A.	2019	Evaluating the Cost-Effective Use of Follow-Up Colonoscopy Based on Screening Findings and Age	Comput. math. methods med. (Computational and mathematical methods in medicine)	10.1155/2019/2476565	2

Knudsen A.B.; Hur C.; Kuntz K.M.; Haug U.; Gazelle G.S.	2012	Effectiveness and cost-effectiveness of once-only screening for colorectal cancer with colonoscopy or computed tomographic colonography	Gastroenterology (Gastroenterology)		6
Knudsen, Amy B.; Hur, Chin; Gazelle, G. Scott; Schrag, Deborah; McFarland, Elizabeth G.; Kuntz, Karen M.	2012	Rescreening of persons with a negative colonoscopy result: results from a microsimulation model	Ann Intern Med (Annals of internal medicine)	10.7326/0003-4819-157-9-201211060-00005	2
Knudsen, Amy B.; Zauber, Ann G.; Rutter, Carolyn M.; Naber, Steffie K.; Doria-Rose, V. Paul; Pabiniak, Chester; Johanson, Colden; Fischer, Sara E.; Lansdorp-Vogelaar, Iris; Kuntz, Karen M.	2016	Estimation of Benefits, Burden, and Harms of Colorectal Cancer Screening Strategies Modeling Study for the US Preventive Services Task Force	JAMA (JAMA)	10.1001/jama.2016.6828	5
Koffijberg H.; Coupe V.M.; Ijzerman M.J.; Greuter M.J.	2017	From evaluation to optimization: Using a meta-model to maximize the benefits of colorectal screening accounting for capacity constraints	Value in Health	10.1016/j.jval.2017.08.2131	6
Kriza C.; Jahn B.; Hassan C.; Kolominsky-Rabas P.L.	2014	Cost-effectiveness simulation of colonography versus colonoscopy in Germany: Is laxative-free colonography cost-effective?	Value Health (Value in health : the journal of the International Society for Pharmacoeconomics and Outcomes Research)	10.1016/j.jval.2014.08.2277	6
Ladabaum U.; Allen J.I.; Wandell M.; Ramsey S.	2011	Screening for colorectal cancer with a blood test: Projected effectiveness and cost-effectiveness of a novel plasma methylated septin-9 DNA (mSEPT9) assay			6

Ladabaum U.; Mannalithara A.	2016	Cost-effectiveness of colorectal cancer (CRC) screening with a multitarget stool DNA Assay (FIT-DNA) vs. Fecal Immunochemical Testing (FIT) or colonoscopy	Am J Gastroenterol (The American journal of gastroenterology)	10.1038/ajg.2014.64		6
Ladabaum U.; Mannalithara A.; Brill J.V.; Levin Z.; Bundorf M.K.	2014	Cost-effectiveness of colorectal cancer screening: Contrasting results for commercial insurance payers vs. medicare	Cancer (Cancer)	10.1002/cncr.29162		6
Ladabaum U.; Mannalithara A.; Mitani A.; Whittemore A.S.; Desai M.	2015	Potential effectiveness and cost-effectiveness of tailoring screening to predicted colorectal cancer risk	Ann Intern Med (Annals of internal medicine)	10.7326/0003-4819-155-2-201107190-00002		6
Ladabaum, Uri; Alvarez-Osorio, Lourdes; Rosch, Thomas; Brueggenjuergen, Bernd	2014	Cost-effectiveness of colorectal cancer screening in Germany: current endoscopic and fecal testing strategies versus plasma methylated Septin 9 DNA	Am J Gastroenterol (The American journal of gastroenterology)	10.1038/ajg.2014.64		2
Ladabaum, Uri; Mannalithara, Ajitha	2016	Comparative Effectiveness and Cost Effectiveness of a Multitarget Stool DNA Test to Screen for Colorectal Neoplasia	Cancer Epidemiol Biomarkers Prev (Cancer epidemiology, biomarkers & prevention : a publication of the American Association for Cancer Research, cosponsored by the American Society of Preventive Oncology)	10.1158/1055-9965.EPI-13-0204		2
Lansdorp-Vogelaar, Iris; Goede, S. Lucas; Bosch, Linda J. W.; Melotte, Veerle; Carvalho, Beatriz; van Engeland, Manon; Meijer, Gerrit A.; Koning, Harry J. de; van Ballegooijen, Marjolein	2018	Cost-effectiveness of High-performance Biomarker Tests vs Fecal Immunochemical Test for Noninvasive Colorectal Cancer Screening	Clin Gastroenterol Hepatol (Clinical gastroenterology and hepatology : the official clinical practice journal of the American Gastroenterological Association)	10.1016/j.cgh.2017.07.011		2

Lee, Kwang-Sig; Park, Eun-Cheol	2016	Cost Effectiveness of Colorectal Cancer Screening Interventions with Their Effects on Health Disparity Being Considered	Cancer Res. Treat. (Cancer research and treatment : official journal of Korean Cancer Association)	10.4143/crt.2015.279	2
Lejeune, Catherine; Dancourt, Vincent; Arveux, Patrick; Bonithon-Kopp, Claire; Faivre, Jean	2010	Cost-effectiveness of screening for colorectal cancer in France using a guaiac test versus an immunochemical test	Int J Technol Assess Health Care (International journal of technology assessment in health care)	10.1017/S026646230999078X	2
Lejeune, Catherine; Le Gleut, Karelle; Cottet, Vanessa; Galimard, Christine; Durand, Gerard; Dancourt, Vincent; Faivre, Jean	2014	The cost-effectiveness of immunochemical tests for colorectal cancer screening	Dig Liver Dis (Digestive and liver disease : official journal of the Italian Society of Gastroenterology and the Italian Association for the Study of the Liver)	10.1016/j.dld.2013.07.018	2
Leleu H.; Vimont A.; Barré S.; Taleb S.; de Bels F.	2019	PCN172 COST-EFFECTIVENESS OF CRC FRENCH SCREENING PROGRAMME FOR AVERAGE RISK INDIVIDUALS	Value Health (Value in health : the journal of the International Society for Pharmacoeconomics and Outcomes Research)	10.1016/j.jval.2019.09.368	6
Leleu H.; Vimont A.; Barré S.; Taleb S.; de Bels F.	2019	PCN397 INCORPORATING RISK SCORES IN CRC SCREENING MODELS: A EXAMPLE FROM THE INCA-PHE CRC MODEL	Value Health (Value in health : the journal of the International Society for Pharmacoeconomics and Outcomes Research)	10.1016/j.jval.2019.09.592	6
Lew, Jie-Bin; St John, D James B; Macrae, Finlay A.; Emery, Jon D.; Ee, Hooi C.; Jenkins, Mark A.; He, Emily; Grogan, Paul; Caruana, Michael; Greuter, Marjolein J. E.; Coupe, Veerle M. H.; Canfell, Karen	2018	Benefits, Harms, and Cost-Effectiveness of Potential Age Extensions to the National Bowel Cancer Screening Program in Australia	Cancer Epidemiol Biomarkers Prev (Cancer epidemiology, biomarkers & prevention : a publication of the American Association for Cancer Research, cosponsored by the American Society of Preventive Oncology)	10.1158/1055-9965.EPI-18-0128	2

Lew, Jie-Bin; St John, D James B; Xu, Xiang-Ming; Greuter, Marjolein J. E.; Caruana, Michael; Cenin, Dayna R.; He, Emily; Saville, Marion; Grogan, Paul; Coupe, Veerle M. H.; Canfell, Karen	2017	Long-term evaluation of benefits, harms, and cost-effectiveness of the National Bowel Cancer Screening Program in Australia: a modelling study	Lancet Public Health (The Lancet. Public health)	10.1016/S2468-2667(17)30105-6		2
Li X.; Wang J.; Chen L.; Xiang W.; Wang D.; Wang Z.; Wu Y.; Wang W.	2016	Opportunistic screening and mass screening for colorectal neoplasm: A cost-effectiveness analysis	Chinese Journal of Gastroenterology	10.3969/j.issn.1008-7125.2016.09.004		7
Li, Y.; Zhu, M.; Klein, R.; Kong, N.	2014	Using a partially observable Markov chain model to assess colonoscopy screening strategies - A cohort study	EUROPEAN JOURNAL OF OPERATIONAL RESEARCH	10.1016/j.ejor.2014.03.004		2
Liang, Qian; Li, Xiaogang; Ye, Guangyao; Hong, Jie; Wang, Jianfeng; Chen, Li; Xiang, Weiyan; Wang, Dengjie; Wu, Yunxia; Wang, Wei; Ge, Zhizheng; Wang, Zhenhua; Fang, Jingyuan	2019	Opportunistic screening versus mass screening for colorectal neoplasms in China: a cost-benefit analysis	International Journal of Clinical and Experimental Medicine			2
Lich, Kristen Hassmiller; Cornejo, David A.; Mayorga, Maria E.; Pignone, Michael; Tangka, Florence K. L.; Richardson, Lisa C.; Kuo, Tzy-Mey; Meyer, Anne-Marie; Hall, Ingrid J.; Smith, Judith Lee; Durham, Todd A.; Chall, Steven A.; Crutchfield, Trisha M.; Wheeler, Stephanie B.	2017	Cost-Effectiveness Analysis of Four Simulated Colorectal Cancer Screening Interventions, North Carolina	Prev Chronic Dis (Preventing chronic disease)	10.5888/pcd14.160158		2
Loomes D.E.; Nguyen T.X.; Kirwin E.; Wang H.; Svenson L.; Fedorak R.N.	2015	A novel urine-based metabolomics screening test for adenomatous polyps is superior to a colonoscopy-based strategy	Gastroenterology (Gastroenterology)			6

Lucidarme, Olivier; Cadi, Mehdi; Berger, Genevieve; Taieb, Julien; Poynard, Thierry; Grenier, Philippe; Beresniak, Ariel	2012	Cost-effectiveness modeling of colorectal cancer: Computed tomography colonography vs colonoscopy or fecal occult blood tests	Eur J Radiol (European journal of radiology)	10.1016/j.ejrad.2011.03.027	2
Ma X.; Li Q.; Ma W.	2012	Cost estimation and control of colorectal cancer screening	Chinese-German Journal of Clinical Oncology	10.1007/s10330-011-0897-1	2
Maceira D.; Espinola N.; Palacios A.	2015	Cost-effectiveness of screening for colorectal cancer in Argentina	Value Health (Value in health : the journal of the International Society for Pharmacoeconomics and Outcomes Research)		6
Matarese V.G.; Bracci E.; Pizzo E.; Vagnoni E.; Feo C.; Stockbrugger R.; Gullini S.	2011	Cost-effectiveness analysis of colorectal screening programme in Italy	Dig Liver Dis (Digestive and liver disease : official journal of the Italian Society of Gastroenterology and the Italian Association for the Study of the Liver)	10.1016/S1590-8658(11)60324-1	6
McFerran E.; Meester R.; De Konnig H.; McVicar D.; Kee F.; Zauber A.G.; Lansdorp-Vogelaar I.	2017	The effect on outcomes of using fecal immunochemical tests (FIT) for surveillance of colorectal adenoma patients within miscan micro-simulation evaluation	Gastrointest Endosc (Gastrointestinal endoscopy)		6
McFerran E.; O'Mahony J.F.	2017	Can ireland's colorectal screening programme save more lives, save money and live within existing colonoscopy capacity limits? findings from the miscan microsimulation model	Value in Health		6

McFerran E.; O'Mahony J.F.	2017	How Ireland's colorectal screening programme could save more lives, save money and stay within existing colonoscopy capacity limits: Evidence from the Miscan microsimulation model	Value in Health	10.1016/j.jval.2017.08.060		6
McLeod, Melissa; Kvizhinadze, Giorgi; Boyd, Matt; Barendregt, Jan; Sarfati, Diana; Wilson, Nick; Blakely, Tony	2017	Colorectal Cancer Screening: How Health Gains and Cost-Effectiveness Vary by Ethnic Group, the Impact on Health Inequalities, and the Optimal Age Range to Screen	Cancer Epidemiol Biomarkers Prev (Cancer epidemiology, biomarkers & prevention : a publication of the American Association for Cancer Research, cosponsored by the American Society of Preventive Oncology)	10.1158/1055-9965.EPI-17-0150		2
Meenan, Richard T.; Coronado, Gloria D.; Petrik, Amanda; Green, Beverly B.	2019	A cost-effectiveness analysis of a colorectal cancer screening program in safety net clinics	Prev Med (Preventive medicine)	10.1016/j.ypmed.2019.01.014		2
Meester R.; Lansdorp-Vogelaar I.; Zauber A.G.; Kaminski M.F.; Holme O.; Dekker E.; Jover R.; Bretthauer M.; Knudsen A.B.; Ladabaum U.	2017	Cost-effectiveness of surveillance guidelines for patients with low-risk or high-risk colorectal adenomas	Gastroenterology (Gastroenterology)			6
Meester R.G.; Doubeni C.A.; Zauber A.G.; Goede L.; Levin T.R.; Corley D.A.; Jemal A.; Lansdorp-Vogelaar I.	2015	Public health impact of achieving 80% colorectal cancer screening rates in the United States by 2018	Gastroenterology (Gastroenterology)			6
Meester, Reinier G. S.; Peterse, Elisabeth F. P.; Knudsen, Amy B.; Weerdt, Anne C. de; Chen, Jennifer C.; Lietz, Anna P.; Dwyer, Andrea; Ahnen, Dennis J.; Siegel, Rebecca L.; Smith, Robert A.; Zauber, Ann G.; Lansdorp-Vogelaar, Iris	2018	Optimizing Colorectal Cancer Screening by Race and Sex: Microsimulation Analysis II to Inform the American Cancer Society Colorectal Cancer Screening Guideline	Cancer (Cancer)	10.1002/cncr.31542		5

Murphy, Jacqueline; Halloran, Stephen; Gray, Alastair	2017	Cost-effectiveness of the faecal immunochemical test at a range of positivity thresholds compared with the guaiac faecal occult blood test in the NHS Bowel Cancer Screening Programme in England	BMJ Open (BMJ open)	10.1136/bmjopen-2017-017186	2
Naber S.K.; Knudsen A.B.; Fischer S.E.; Carolyn R.; Pabiniak C.; Kuntz K.; Zauber A.G.; Lansdorp-Vogelaar I.	2016	Comparative cost-effectiveness of multi-targeted stool DNA testing in the medicare population	Gastroenterology (Gastroenterology)		6
Naber, Steffie K.; Kundu, Suman; Kuntz, Karen M.; Dotson, W. David; Williams, Marc S.; Zauber, Ann G.; Calonge, Ned; Zallen, Doris T.; Ganiats, Theodore G.; Webber, Elizabeth M.; Goddard, Katrina A. B.; Henrikson, Nora B.; van Ballegooijen, Marjolein; Janssens, A Cecile J W; Lansdorp-Vogelaar, Iris	2020	Cost-Effectiveness of Risk-Stratified Colorectal Cancer Screening Based on Polygenic Risk: Current Status and Future Potential	JNCI cancer spectr. (JNCI cancer spectrum)	10.1093/jncics/pkz086	2
Nahkur O.; Männik A.; Suuroja T.; Juus E.; Võrno T.; Reile R.; Kiivet R.-A.	2015	The cost-effectiveness of screening for colorectal cancer	Eesti Arst		7
Neubauer G.; Minartz C.	2010	Costs and benefits of colorectal cancer prevention	Onkologe	10.1007/s00761-010-1905-8	5
Okafor P.N.; Erim D.O.; Talwalkar J.A.	2015	A cost-effectiveness analysis of early colorectal cancer screening using multi-target stool DNA and optical colonoscopy in African Americans	Am J Gastroenterol (The American journal of gastroenterology)	10.1038/ajg.2015.271	6
Orsak G.; Miller A.; Allen C.M.; Singh K.P.; McGaha P.	2020	Return on Investment of Free Colorectal Cancer Screening Tests in a Primarily Rural Uninsured or Underinsured Population in Northeast Texas	PharmacoEconomics - Open	10.1007/s41669-019-0147-y	5

Palimaka, Stefan; Blackhouse, Gord; Goeree, Ron	2015	Colon Capsule Endoscopy for the Detection of Colorectal Polyps: An Economic Analysis	Ont Health Technol Assess Ser (Ontario health technology assessment series)		2
Pereira A.; Areia M.; Dinis-Ribeiro M.	2016	Cost utility analysis of genetic polymorphisms testing for colorectal cancer: Is there a role for genetic tailoring in colorectal cancer prevention?	Gastroenterology (Gastroenterology)		6
Peterse E.F.; Meester R.; de Jonge L.; Alarid-Escudero F.; Zauber A.G.; Lansdorp-Vogelaar I.	2019	COMPARING THE COST-EFFECTIVENESS OF NEW COLORECTAL CANCER SCREENING TESTS	Gastroenterology (Gastroenterology)	10.1016/S0016-5085(19)36826-X	6
Peterse E.F.; Meester R.; Gini A.; Berger F.G.; Zauber A.G.; Lansdorp-Vogelaar I.	2017	Cost-effectiveness of waiving all copayments for colorectal cancer screening among medicare beneficiaries	Gastroenterology (Gastroenterology)		6
Peterse E.F.; Meester R.; Siegel R.; Chen J.; Dwyer A.; Ahnen D.; Smith R.; Zauber A.G.; Lansdorp-Vogelaar I.	2018	Colorectal Cancer Screening Initiation at Age 45 Years: A Microsimulation Analysis to Address the Rising Incidence in Young Adults	Gastroenterology (Gastroenterology)	10.1016/S0016-5085(18)30692-9	6
Peterse E.F.P.; Meester R.G.S.; Siegel R.L.; Chen J.C.; Dwyer A.; Ahnen D.J.; Smith R.A.; Zauber A.G.; Lansdorp-Vogelaar I.	2018	The impact of the rising colorectal cancer incidence in young adults on the optimal age to start screening in the US: A microsimulation analysis	J. glob. oncol. (Journal of global oncology)	10.1200/jgo.18.34900	6
Peterse, Elisabeth F. P.; Meester, Reinier G. S.; Siegel, Rebecca L.; Chen, Jennifer C.; Dwyer, Andrea; Ahnen, Dennis J.; Smith, Robert A.; Zauber, Ann G.; Lansdorp-Vogelaar, Iris	2018	The Impact of the Rising Colorectal Cancer Incidence in Young Adults on the Optimal Age to Start Screening: Microsimulation Analysis I to Inform the American Cancer Society Colorectal Cancer Screening Guideline	Cancer (Cancer)	10.1002/cncr.31543	5

Phisalprapa, Pochamana; Supakankunti, Siripen; Chaiyakunapruk, Nathorn	2019	Cost-effectiveness and budget impact analyses of colorectal cancer screenings in a low- and middle-income country: example from Thailand	J Med Econ (Journal of medical economics)	10.1080/13696998.2019.1674065		2
Pignone, Michael P.; Flitcroft, Kathy L.; Howard, Kirsten; Trevena, Lyndal J.; Salkeld, Glenn P.; St John, D. James B.	2011	Costs and cost-effectiveness of full implementation of a biennial faecal occult blood test screening program for bowel cancer in Australia	Med J Aust (The Medical journal of Australia)	10.5694/j.1326-5377.2011.tb03766.x		2
Pil, L.; Fobelets, M.; Putman, K.; Trybou, J.; Annemans, L.	2016	Cost-effectiveness and budget impact analysis of a population-based screening program for colorectal cancer	EUR. J. INTERN. MED. (European journal of internal medicine)	10.1016/j.ejim.2016.03.031		2
Pinzón Flórez C.E.; Gamboa O.A.; Murillo moreno R.; Rosselli D.	2012	Cost-effectiveness of screening and early detection strategies for colorectal cancer in Colombia	Value Health (Value in health : the journal of the International Society for Pharmacoeconomics and Outcomes Research)	10.1016/j.jval.2012.03.370		6
Pinzon Florez C.E.; Rosselli D.; Gamboa Garay O.A.	2012	Cost-effectiveness analysis of screening strategies for colorectal cancer in Colombia	Value Health Reg Issues (Value in health regional issues)	10.1016/j.vhri.2012.09.006		7
Pizzo E.; Bracci E.; Vagnoni E.; Wilschut J.; Van Ballegooijen M.	2010	Cost-effectiveness analysis of a FOBT-based colorectal cancer screening program	Value Health (Value in health : the journal of the International Society for Pharmacoeconomics and Outcomes Research)			6
Pohl, Heiko; Robertson, Douglas J.	2010	Colorectal Cancers Detected After Colonoscopy Frequently Result From Missed Lesions	Clin Gastroenterol Hepatol (Clinical gastroenterology and hepatology : the official clinical practice journal of the American Gastroenterological Association)	10.1016/j.cgh.2010.06.028		5

Prakash, Meher K.; Lang, Brian; Heinrich, Henriette; Valli, Piero V.; Bauerfeind, Peter; Sonnenberg, Amnon; Beerenwinkel, Niko; Misselwitz, Benjamin	2017	CMOST: an open-source framework for the microsimulation of colorectal cancer screening strategies	BMC Med Inf Decis Mak (BMC medical informatics and decision making)	10.1186/s12911-017-0458-9		5
Pyenson, Bruce; Pickhardt, Perry J.; Sawhney, Tia Goss; Berrios, Michele	2015	Medicare cost of colorectal cancer screening: CT colonography vs. optical colonoscopy	Abdom Imaging (Abdominal imaging)	10.1007/s00261-015-0538-1		5
Rabeneck, Linda; Lansdorp-Vogelaar, Iris	2015	Assessment of a cancer screening program	Baillieres Best Pract Res Clin Gastroenterol (Best practice & research. Clinical gastroenterology)	10.1016/j.bpg.2015.09.009		5
Ralaidovy A.H.; Gopalappa C.; Ilbawi A.; Pretorius C.; Lauer J.A.	2018	Cost-effective interventions for breast cancer, cervical cancer, and colorectal cancer: New results from WHO-CHOICE 11 Medical and Health Sciences 1117 Public Health and Health Services 11 Medical and Health Sciences 1112 Oncology and Carcinogenesis	Cost Eff Resour Alloc (Cost effectiveness and resource allocation : C/E)	10.1186/s12962-018-0157-0		6
Ramirez-Cervantes K.L.; Landy R.; Hounsome N.; Gillman M.S.; Troche J.M.R.; Marquez J.A.R.; Gallo V.	2018	Costs and Benefits of Implementing a Colorectal Cancer Screening Programme in Mexico	Gastroenterology (Gastroenterology)	10.1016/S0016-5085(18)32114-0		6
Roberts-Thomson, Ian C.; Lung, Thomas	2018	Cost-effective options for the prevention and management of gastrointestinal and liver disease in the Asia-Pacific region	J Gastroenterol Hepatol (Journal of gastroenterology and hepatology)	10.1111/jgh.13925		5

Romero M.; Vasquez Melo E.C.; Acero Acero G.; Marino C.	2018	COST-EFFECTIVENESS ANALYSIS OF THE USE OF COLONOSCOPY AS A SCREENING METHOD FOR COLON CANCER IN PATIENTS OVER 50 YEARS OF AGE ON MANDATORY HEALTH INSURANCE	Value Health (Value in health : the journal of the International Society for Pharmacoeconomics and Outcomes Research)	10.1016/j.jval.2018.09.240		6
Rose, Johnie; Augestad, Knut Magne; Kong, Chung Yin; Meropol, Neal J.; Kattan, Michael W.; Hong, Qingqing; An, Xuebei; Cooper, Gregory S.	2014	A simulation model of colorectal cancer surveillance and recurrence	BMC Med Inf Decis Mak (BMC medical informatics and decision making)	10.1186/1472-6947-14-29		2
Ruggeri M.; Coretti S.; Di Bidino R.; Marcellusi A.; Mennini F.; Cicchetti A.	2012	Economic evaluation of a colorectal cancer screening program: Indirect evidence on long-term outcomes and economic sustainability in a regional perspective	PharmacoEconomics - Italian Research Articles			7
Rutter, Carolyn M.; Kim, Jane J.; Meester, Reinier G. S.; Sprague, Brian L.; Burger, Emily A.; Zauber, Ann G.; Ergun, Mehmet Ali; Campos, Nicole G.; Doubeni, Chyke A.; Trentham-Dietz, Amy; Sy, Stephen; Alagoz, Oguzhan; Stout, Natasha; Lansdorp-Vogelaar, Iris; Corley, Douglas A.; Tosteson, Anna N. A.	2018	Effect of Time to Diagnostic Testing for Breast, Cervical, and Colorectal Cancer Screening Abnormalities on Screening Efficacy: A Modeling Study	Cancer Epidemiol Biomarkers Prev (Cancer epidemiology, biomarkers & prevention : a publication of the American Association for Cancer Research, cosponsored by the American Society of Preventive Oncology)	10.1158/1055-9965.EPI-17-0378		5
Rutter, Carolyn M.; Knudsen, Amy B.; Marsh, Tracey L.; Doria-Rose, V. Paul; Johnson, Eric; Pabiniak, Chester; Kuntz, Karen M.; van Ballegooijen, Marjolein; Zauber, Ann G.; Lansdorp-Vogelaar, Iris	2016	Validation of Models Used to Inform Colorectal Cancer Screening Guidelines: Accuracy and Implications	Med Decis Making (Medical decision making : an international journal of the Society for Medical Decision Making)	10.1177/0272989X15622642		5
Rutter, Carolyn M.; Miglioretti, Diana L.; Savarino, James E.	2011	Evaluating risk factor assumptions: a simulation-based approach	BMC Med Inf Decis Mak (BMC medical informatics and decision making)	10.1186/1472-6947-11-55		5

Sadeghi S.; Quinn D.I.; Lenz H.-J.; Barzi A.	2016	FOBT compared to FIT in reducing colorectal cancer (CRC) incidence	Journal of Clinical Oncology		6
Saengow, Udomsak; Birch, Stephen; Geater, Alan; Chongsuwiwatvong, Virasakdi	2018	Willingness to Pay for Colorectal Cancer Screening and Effect of Copayment in Southern Thailand	Asian Pac J Cancer Prev (Asian Pacific journal of cancer prevention : APJCP)		2
Saini S.D.; Schoenfeld P.S.; Vijan S.	2011	The unintended consequences of efforts to improve colonoscopy sensitivity	Gastroenterology (Gastroenterology)	10.1016/S0016-5085(11)62300-7	6
Sakamaki H.; Tajiri H.; Inoue S.	2013	Cost-effectiveness analysis of capsule endoscopy in screening for colorectal cancer in Japan	Value Health (Value in health : the journal of the International Society for Pharmacoeconomics and Outcomes Research)	10.1016/j.jval.2013.08.526	6
Sat-Muñoz D.; Duran-Anguiano O.; Palomares-Chacon U.; Gonzalez-Barba F.; Martinez-Herrera B.; Gómez-Sánchez E.; Bañuelos-Rizo M.; Balderas-Peña L.	2018	Cost-Utility Analysis of Colorectal Cancer Treatment in A Third Level Public Hospital in Mexico	Value Health (Value in health : the journal of the International Society for Pharmacoeconomics and Outcomes Research)	10.1016/j.jval.2018.07.130	6
Schlander, Michael; Cheng, Chih-Yuan; Ran, Tao	2018	The health economics of cancer screening in Germany: Which population-based interventions are cost-effective?	Bundesgesundheitsblatt Gesundheitsforschung Gesundheitsschutz (Gesundheitsokonomie der Krebsfruherkennung in Deutschland: Welche Interventionen sind kosteneffektiv bei bevolkerungsweiter Umsetzung?)	10.1007/s00103-018-2839-3	5
Schroy P.; Berger B.M.; Dinh T.A.	2015	Multi-target stool DNA-based colorectal cancer screening: Modeling the impact of inter-test interval on clinical effectiveness	Am J Gastroenterol (The American journal of gastroenterology)	10.1038/ajg.2015.278	6

Sekiguchi M.; Matsuda T.; Igarashi A.; Matsumoto M.; Sakamoto T.; Nakajima T.; Kakugawa Y.; Saito Y.	2015	Cost-effectiveness analysis on surveillance following negative colonoscopy in populationbased colorectal cancer screening	United European Gastroenterol. j. (United European gastroenterology journal)	10.1177/2050640615601611		6
Sekiguchi M.; Matsuda T.; Igarashi A.; Matsumoto M.; Sakamoto T.; Nakajima T.; Kakugawa Y.; Saito Y.	2015	What is the optimal use of total colonoscopy in colorectal cancer screening? A cost-effectiveness analysis using Japanese data	Gastroenterology (Gastroenterology)			6
Sekiguchi M.; Matsuda T.; Matsumoto M.; Sakamoto T.; Otake Y.; Nakajima T.; Kakugawa Y.; Saito Y.	2012	Cost-effectiveness of total colonoscopy for colorectal cancer screening in Japan	Gastrointest Endosc (Gastrointestinal endoscopy)	10.1016/j.gie.2012.03.1353		6
Sekiguchi, Masau; Igarashi, Ataru; Matsuda, Takahisa; Matsumoto, Minori; Sakamoto, Taku; Nakajima, Takeshi; Kakugawa, Yasuo; Yamamoto, Seiichiro; Saito, Hiroshi; Saito, Yutaka	2016	Optimal use of colonoscopy and fecal immunochemical test for population-based colorectal cancer screening: a cost-effectiveness analysis using Japanese data	Jpn J Clin Oncol (Japanese journal of clinical oncology)	10.1093/jjco/hyv186		2
Senore C.; Hassan C.; Regge D.; Pagano E.; Iussich G.; Segnan N.	2015	Cost-effectiveness of organized programs for colorectal cancer screening: Sigmoidoscopy and immunochemical faecal occult blood test	Gastroenterology (Gastroenterology)			6
Shah J.; Bhattacharya R.; Sansgiry S.S.	2010	Is fecal immunochemical test economically the best screening test for colorectal cancer?	Pharmacotherapy (Pharmacotherapy)			6
Sharaf R.; Ladabaum U.	2011	Comparative effectiveness and cost-Effectiveness of screening colonoscopy vs. Sigmoidoscopy and alternative strategies 2011 ACG presidential poster	Am J Gastroenterol (The American journal of gastroenterology)	10.1038/ajg.2011.336_13		6

Sharma, T.	2020	Analysis of the effectiveness of two noninvasive fecal tests used to screen for colorectal cancer in average-risk adults	Public Health (Public health)	10.1016/j.puhe.2020.01.021		2
Sharp, Linda; Tilson, Lesley; Whyte, Sophie; Ceilleachair, Alan O.; Walsh, Cathal; Usher, Cara; Tappenden, Paul; Chilcott, James; Staines, Anthony; Barry, Michael; Comber, Harry	2013	Using resource modelling to inform decision making and service planning: the case of colorectal cancer screening in Ireland	BMC Health Serv Res (BMC health services research)	10.1186/1472-6963-13-105		5
Shinkins B.; Smith A.; King N.; Davies A.; Wilson I.	2017	Early economic modelling of EMI-137 to improve the detection rate of polyps in patients with increased risk of colorectal cancer	United European Gastroenterol. j. (United European gastroenterology journal)	10.1177/2050640617725676		6
Shirley L.; Nightingale J.M.	2013	Establishing the role of CT colonography within the Bowel Cancer Screening Programme	Radiography	10.1016/j.radi.2013.03.003		5
Smith, David H.; O'Keeffe Rosetti, Maureen; Mosen, David M.; Rosales, A. Gabriela; Keast, Erin; Perrin, Nancy; Feldstein, Adrianne C.; Levin, Theodore R.; Liles, Elizabeth G.	2019	Balancing Adherence and Expense: The Cost-Effectiveness of Two-Sample vs One-Sample Fecal Immunochemical Test	Popul Health Manag (Population health management)	10.1089/pop.2018.0008		2
Sobhani, Iradj; Alzahouri, Kazem; Ghout, Idir; Charles, Delchier Jean; Durand-Zaleski, Isabelle	2011	Cost-Effectiveness of Mass Screening for Colorectal Cancer: Choice of Fecal Occult Blood Test and Screening Strategy	Dis Colon Rectum (Diseases of the colon and rectum)	10.1007/DCR.0b013e31820fd2bc		2
Song, Li-Peng; Wang, Hao-Yu	2016	Modeling and Control of Colorectal Cancer	PLoS ONE (PloS one)	10.1371/journal.pone.0161349		2
Subramanian, Sujha; Bobashev, Georgiy; Morris, Robert J.	2010	When Budgets Are Tight, There Are Better Options Than Colonoscopies For Colorectal Cancer Screening	Health Aff (Millwood) (Health affairs (Project Hope))	10.1377/hlthaff.2008.0898		2

Subramanian, Sujha; Bobashev, Georgiy; Morris, Robert J.; Hoover, Sonja	2017	Personalized medicine for prevention: can risk stratified screening decrease colorectal cancer mortality at an acceptable cost?	Cancer Causes Control (Cancer causes & control : CCC)	10.1007/s10552-017-0864-4		2
Sweet, Alison; Lee, David; Gairy, Kerry; Phiri, Denver; Reason, Timothy; Lock, Kevin	2011	The impact of CT colonography for colorectal cancer screening on the UK NHS: costs, healthcare resources and health outcomes	Appl Health Econ Health Policy (Applied health economics and health policy)	10.2165/11588110-000000000-00000		2
Taksler, Glen B.; Perzynski, Adam T.; Kattan, Michael W.	2017	Modeling Individual Patient Preferences for Colorectal Cancer Screening Based on Their Tolerance for Complications Risk	Med Decis Making (Medical decision making : an international journal of the Society for Medical Decision Making)	10.1177/0272989X16679161		5
Tran, B.; Keating, C. L.; Ananda, S. S.; Kosmider, S.; Jones, I.; Croxford, M.; Field, K. M.; Carter, R. C.; Gibbs, P.	2012	Preliminary analysis of the cost-effectiveness of the National Bowel Cancer Screening Program: demonstrating the potential value of comprehensive real world data	Intern Med J (Internal medicine journal)	10.1111/j.1445-5994.2011.02585.x		2
van Ballegooijen, Marjolein; Rutter, Carolyn M.; Knudsen, Amy B.; Zauber, Ann G.; Savarino, James E.; Lansdorp-Vogelaar, Iris; Boer, Rob; Feuer, Eric J.; Habbema, J. Dik F.; Kuntz, Karen M.	2011	Clarifying Differences in Natural History between Models of Screening: The Case of Colorectal Cancer	Med Decis Making (Medical decision making : an international journal of the Society for Medical Decision Making)	10.1177/0272989X11408915		2
van der Meulen, Miriam P.; Lansdorp-Vogelaar, Iris; Goede, S. Lucas; Kuipers, Ernst J.; Dekker, Evelien; Stoker, Jaap; van Ballegooijen, Marjolein	2018	Colorectal Cancer: Cost-effectiveness of Colonoscopy versus CT Colonography Screening with Participation Rates and Costs	Radiology (Radiology)	10.1148/radiol.2017162359		2
van der Steen, Alex; Knudsen, Amy B.; van Hees, Frank; Walter, Gailya P.; Berger, Franklin G.; Daguise, Virginie G.; Kuntz, Karen M.; Zauber, Ann G.; van Ballegooijen, Marjolein; Lansdorp-Vogelaar, Iris	2015	Optimal colorectal cancer screening in states' low-income, uninsured populations-the case of South Carolina	Health Serv Res (Health services research)	10.1111/1475-6773.12246		2

van Hees, Frank; Saini, Sameer D.; Lansdorp-Vogelaar, Iris; Vijan, Sandeep; Meester, Reinier G. S.; Koning, Harry J. de; Zauber, Ann G.; van Ballegooijen, Marjolein	2015	Personalizing Colonoscopy Screening for Elderly Individuals Based on Screening History, Cancer Risk, and Comorbidity Status Could Increase Cost Effectiveness	Gastroenterology (Gastroenterology)	10.1053/j.gastro.2015.07.042	2
van Hees, Frank; Zauber, Ann G.; Klabunde, Carrie N.; Goede, S. Luuk; Lansdorp-Vogelaar, Iris; van Ballegooijen, Marjolein	2014	The Appropriateness of More Intensive Colonoscopy Screening Than Recommended in Medicare Beneficiaries A Modeling Study	JAMA Intern Med (JAMA internal medicine)	10.1001/jamainternmed.2014.3889	2
van Rossum, Leo G M; van Rijn, Anne F.; Verbeek, Andre L. M.; van Oijen, Martijn G H; Laheij, Robert J. F.; Fockens, Paul; Jansen, Jan B M J; Adang, Eddy M. M.; Dekker, Evelien	2011	Colorectal cancer screening comparing no screening, immunochemical and guaiac fecal occult blood tests: a cost-effectiveness analysis	Int J Cancer (International journal of cancer)	10.1002/ijc.25530	2
Vleugels J.; Greuter M.J.E.; Hazewinkel Y.; Coupé V.M.H.; Dekker E.	2016	Implementation of an optical diagnosis strategy saves costs and does not impair clinical outcomes of a fit-based CRC screening programme	United European Gastroenterol. j. (United European gastroenterology journal)	10.1177/2050640616663689	2
Wang, Zhen-Hua; Gao, Qin-Yan; Fang, Jing-Yuan	2012	Repeat Colonoscopy Every 10 Years or Single Colonoscopy for Colorectal Neoplasm Screening in Average-risk Chinese: A Cost-effectiveness Analysis	Asian Pac J Cancer Prev (Asian Pacific journal of cancer prevention : APJCP)	10.7314/APJCP.2012.13.5.1761	2

Westwood, Marie; Corro Ramos, Isaac; Lang, Shona; Luyendijk, Marianne; Zaim, Remziye; Stirk, Lisa; Al, Maiwenn; Armstrong, Nigel; Kleijnen, Jos	2017	Faecal immunochemical tests to triage patients with lower abdominal symptoms for suspected colorectal cancer referrals in primary care: a systematic review and cost-effectiveness analysis	Health Technol Assess (Health technology assessment (Winchester, England))	10.3310/hta21330	2
Whyte, Sophie; Walsh, Cathal; Chilcott, Jim	2011	Bayesian calibration of a natural history model with application to a population model for colorectal cancer	Med Decis Making (Medical decision making : an international journal of the Society for Medical Decision Making)	10.1177/0272989X10384738	2
Wilschut J.; Hol L.; Dekker E.; Jansen J.B.; Van Leerdam M.E.; Lansdorp-Vogelaar I.; Kuipers E.J.; Habbema J.D.; Van Ballegooijen M.	2010	Quantitative immunochemical fecal occult blood screening under a colonoscopy constraint: A Higher cut-off level, a smaller age range or a longer screening interval? A cost-effectiveness analysis	Gastroenterology (Gastroenterology)		6
Wilschut, Janneke A.; Habbema, J. Dik F.; van Leerdam, Monique E.; Hol, Lieke; Lansdorp-Vogelaar, Iris; Kuipers, Ernst J.; van Ballegooijen, Marjolein	2011	Fecal occult blood testing when colonoscopy capacity is limited	J Natl Cancer Inst (Journal of the National Cancer Institute)	10.1093/jnci/djr385	2
Wohl, Pavel; Bednarik, Michal; Wohl, Petr; Cervenka, Milan; Spicak, Julius	2011	Comparison of various strategies for colorectal cancer screening tests	Eur J Gastroenterol Hepatol (European journal of gastroenterology & hepatology)	10.1097/MEG.0b013e3283491438	6
Wong M.C.; Ching J.; Chan V.C.; Lam T.Y.; Luk A.K.; Ng S.C.; Ng S.S.; Sung J.J.Y.	2014	The comparative cost-effectiveness of faecal immunochemical tests vs. screening colonoscopy in the detection of neoplastic lesions	Gastrointest Endosc (Gastrointestinal endoscopy)	10.1016/j.gie.2014.02.067	6

Wong, Carlos K. H.; Lam, Cindy L. K.; Wan, Y. F.; Fong, Daniel Y. T.	2015	Cost-effectiveness simulation and analysis of colorectal cancer screening in Hong Kong Chinese population: comparison amongst colonoscopy, guaiac and immunologic fecal occult blood testing	BMC Cancer (BMC cancer)	10.1186/s12885-015-1730-y	2
Wong, Martin C. S.; Ching, Jessica Y. L.; Chan, Victor C. W.; Sung, Joseph J. Y.	2015	The comparative cost-effectiveness of colorectal cancer screening using faecal immunochemical test vs. colonoscopy	Sci. rep. (Scientific reports)	10.1038/srep13568	2
Worthington, Joachim; Lew, Jie-Bin; Feletto, Eleonora; Holden, Carol A.; Worthley, Daniel L.; Miller, Caroline; Canfell, Karen	2020	Improving Australian National Bowel Cancer Screening Program outcomes through increased participation and cost-effective investment	PLoS ONE (PloS one)	10.1371/journal.pone.0227899	2
Yu T.M.; Tradodnsky A.; Tang J.; Arnold R.	2018	Cost-Effectiveness of Adding Endocuff® to Colonoscopies for Interval Colorectal Cancer Screening	Gastroenterology (Gastroenterology)	10.1016/S0016-5085(18)31771-2	6
Yu, Tiffany M.; Tradonsky, Alison; Tang, Jun; Arnold, Renee Jg	2019	Cost-effectiveness of adding Endocuff R to standard colonoscopies for interval colorectal cancer screening	ClinicoEcon. outcomes res. (ClinicoEconomics and outcomes research : CEOR)	10.2147/CEOR.S201328	2
Zauber A.G.; Knudsen A.B.; Carolyn R.; Naber S.K.; Doria-Rose P.; Pabiniak C.; Fischer S.E.; Lansdorp-Vogelaar I.; Kuntz K.	2016	Evaluating the benefits and harms of colorectal cancer screening strategies: A collaborative modeling approach to inform the us preventive services task force	Gastroenterology (Gastroenterology)		6
Zauber, Ann G.	2010	Cost-effectiveness of colonoscopy	Gastrointest Endosc Clin N Am (Gastrointestinal endoscopy clinics of North America)	10.1016/j.giec.2010.07.008	5

Zheng P.; Dinh T.	2014	A novel colorectal cancer model with sessile serrated adenoma pathway to evaluate the cost-effectiveness of various screening strategies	Value Health (Value in health : the journal of the International Society for Pharmacoeconomics and Outcomes Research)	10.1016/j.jval.2014.03.483	6

Reasons for exclusion:

1: not general population
2: no sigmoidoscopy
3: no adequate comparator
4: only non-comparable outcome
5: no (full) ecomomic analysis
6: abstract publication
7: not English or German
