# Supplementary material for: Is there a place for sigmoidoscopy in colorectal cancer screening? A systematic review and critical appraisal of cost-effectiveness models
Source: PLoS One. 2023 Aug 18;18(8):e0290353. doi: 10.1371/journal.pone.0290353 (PMC10438011; doi:10.1371/journal.pone.0290353)
Supplement: S1 File — (PDF) [file pone.0290353.s005.pdf]

Table A1: Overview of screening strategies with sigmoidoscopy

	in combination with	n / 25 studies	Barré et al. 2020	Barzi et al. 2017	Dan et al. 2012	Hassan et al. 2011	Heitman et al. 2010	Kingsley et al. 2016	Knudsen et al. 2010	Ladabaum et al. 2013	Ladabaum et al. 2018	Ladabaum et al. 2019	Lam et al. 2015	Landsdorp-Vogelaar et al. 2010	Lee et al. 2010	Lew et al. 2018	Melnitchouk et al. 2018	Naber et al. 2019	Senore et al. 2019	Sharaf & Ladabaum 2013	Sharp et al. 2012	Telford et al. 2010	Tuan Dinh et al. 2013	Van Hees et al. 2014	Vanness et al. 2011	Whyte et al. 2012	Wong et al. 2016
**Sig Once**		**7**			@60											@60			@58	@60	@60 [@55]			@76, 80,85 or 90		@55	
**Sig 5y**		**15**		x	x	x	x	x	x	x	x		x	x				x		x		x	x				x[1]
**Sig 10y**		**6**	x			x							x		x	x										@55,65	
**Sig 5y**	**FOBT 1y**	**9**		x					x	x	x		x	x			x					x			x		
	**FOBT 2y**	**1**		x																							
	**FOBT 3y**	**1**												x													
	**FIT 1y**	**8**		x	x				x	x	x		x	x											x		
	**FIT 2y**	**2**		x										x													
	**FIT 3y**	**1**																		x							
	**COL**	**1**																									- Sig @50+55; Col @60+70 - Sig @50, 55, 60, 65; Col Once @70 [2]
**Sig 10y**	**FOBT 1y**	**2**											x					x									
	**FIT 1y**	**3**											x					x					x				
**Sig Once**																											
**@45**	**COL 10y**	**1**										(50-75)															
	**FIT 1y**	**1**										(50-75)															
**@55**	**FOBT 2y**	**1**																								(66-74)	
	**FIT 2y**	**2**														(60-74)										(56-74 / 60-74 / 66-74)	
	**FIT @60,65,70**	**1**																								x	

Sig: sigmoidoscopy
COL: colonoscopy
FOBT: guaiac-based fecal occult blood test
FIT: fecal immunochemical tests

[1] Both male and female subjects received Sig every 5 years OR Sig for each woman at 50-, 55-, 60-, 65-, and 70-year old; male subjects received colonoscopy at 50-, 60-, and 70-year old
[2] Only Women; Men Col @ 50, 60, 70

Table 5 Incremental cost, life years gained, quality-adjusted life years and incremental cost-effectiveness ratios of 10-yearly and once-only sigmoidoscopy compared no screening or 10-yearly colonoscopy (in 2019 $)

				**10-yearly sigmoidoscopy**								**Once-only sigmoidoscopy**				
				vs. no screening				vs. 10-yearly colonoscopy				vs. no screening				
**Author, Year**	**Country**	**Model Name**	**Screening Period**	ΔCost	LYG	ΔQALY	ICER	ΔCost	LYG	ΔQALY	ICER	@Age	ΔCost	LYG	ΔQALY	ICER
Barré 2020	FR	Microsimulation	50-74	30.051		8.700	3.454	-	-	-	-	-	-	-	-	-
Dan 2012	SG	Markov	50-75	-	-	-	-	-	-	-	-	@60	85.658		0.003	28,552.726
Hassan 2011	FR	Markov	50-75	99.849	0.037		2,694.992	-341.421	-0.010		32,547.253	-	-	-	-	-
Lee 2010	UK	Markov	60-69	-	-	-	-	-58.110	-0.003	-0.004	19,370.136 /LYG 14,527.602 /QALY	-	-	-	-	-
Lew 2018	AU	Policy1-Bowel	50-74	267.861	0.004		68,682.310	-1.539	-0.003		481.072	@60	89.287	0.002		38,820.436
Senore 2019	IT	Markov	58-70	-	-	-	-	-	-	-	-	@58	-10.567	0.012		**CS**
Sharaf 2013	US	Markov	50-80	-	-	-	-	-	-	-	-	@60	-321.249		0.026	**CS**
Sharp 2012	IE	Markov	55-74	-	-	-	-	-	-	-	-	@60	2.410		0.005	481.947
				-	-	-	-	-	-	-	-	@55	14.458		0.007	2,065.488
Van Hees 2014	US	MISCAN	76-90	-	-	-	-	-	-	-	-	@76	439.000		53.900	8.145
				-	-	-	-	-	-	-	-	@80	764.000		38.600	19.793
				-	-	-	-	-	-	-	-	@85	1,251.000		14.300	87.483
				-	-	-	-	-	-	-	-	@90	1,580.000		-1.000	**D**
Whyte 2012	UK	Markov	55-74	37.537	0.039	0.033	962.477/LYG 1,137.473 /QALY	-	-	-	-	@55	24.190	0.025	0.021	967.611/LYG 1,130.386 /QALY

FR: France; US: United States of America; SG: Singapore; CA: Canada; HK: Hong Kong; UK: United Kingdom; AU: Australia; IT: Italy; IE: Ireland; UA: Ukraine
CS: cost saving, when a strategy was less costly and equally or more effective
D: dominant, when a strategy was equally or more costly and less effective
LYG: life years gained
QALY: quality-adjusted life years
ICER: incremental cost-effectiveness ratio
n/a: not applicable

Table A2 Incremental cost, life years gained, quality-adjusted life years and incremental cost-effectiveness ratios of 5-yearly sigmoidoscopy compared with biennial strategies (in 2019 $)

				5-yearly sigmoidoscopy							
				vs. biennial FOBT				vs. biennial FIT			
Author, Year	Country	Model Name	Screening Period	ΔCost	LYG	ΔQALY	ICER	ΔCost	LYG	ΔQALY	ICER
Barzi 2017	US	Markov	50-75	98.157	0.003		32,718.926	-44.224	0.006		**CS**
Hassan 2011	FR	Markov	50-75	157.021	0.013		11,931.710	64.419	0.000		**D**
Telford 2010	CA	Markov	50-75	260.726		0.028	9,311.636[a]				

FR: France; US: United States of America; CA: Canada
CS: cost saving, when a strategy was less costly and equally or more effective
D: dominant, when a strategy was equally or more costly and less effective
LYG: life years gained
QALY: quality-adjusted life years
ICER: incremental cost-effectiveness ratio
FOBT: guaiac-based fecal occult blood test
FIT: fecal immunochemical tests

[a] Low-Sensitivity Test

Table A4 Incremental cost, life years gained, quality-adjusted life years and incremental cost-effectiveness ratios of 5-yearly sigmoidoscopy combined with other screening measures tests compared with no screening (in 2019 $)

Author, Year	Country	Model Name	Screening Period	5-yearly sigmoidoscopy + annual FOBT vs. No Screening				5-yearly sigmoidoscopy + annual FIT vs. No Screening				5-yearly sigmoidoscopy + biennial/triennial FOBT vs. No Screening				5-yearly sigmoidoscopy + biennial/triennial FIT vs. No Screening				5-yearly sigmoidoscopy + colonoscopy vs. No Screening			
				ΔCost	LYG	ΔQALY	ICER	ΔCost	LYG	ΔQALY	ICER	ΔCost	LYG	ΔQALY	ICER	ΔCost	LYG	ΔQALY	ICER	ΔCost	LYG	ΔQALY	ICER
Barzi 2017	US	Markov	50-75	-264.268	0.012		**CS**	-83.056	0.009		**CS**	-309.571	0.014		**CS**[e]	-171.505	0.012		**CS**[e]	-	-	-	-
Dan 2012	SG	Markov	50-75	-	-	-	-	363.142		0.007	51,877.488	-	-	-	-	-	-	-	-	-	-	-	-
Knudsen 2010	US	CRC-SPIN	65-80	-395.800<br>-382.236	0.093<br>0.100		**CS**[b]<br>**CS**[c]	-256.468	0.099		**CS**	-	-	-	-	-	-	-	-	-	-	-	-
Vanness 2011	US	CRC-SPIN	50-80	411.829	0.113		3,644.503	541.296	0.113		4,790.229	-	-	-	-	-	-	-	-	-	-	-	-
Knudsen 2010	US	MISCAN	65-80	93.710<br>178.788	0.085<br>0.088		1,103.764[b]<br>2,031.681[c]	378.537	0.088		4,296.677	-	-	-	-	-	-	-	-	-	-	-	-
Lansdorp-Vogelaar 2010[a]	US	MISCAN	65-80	-	-	-	-	-	-	-	-	103.026	0.083		1239.786[f]	268.892	0.083		3243.572[f]	-	-	-	-
Vanness 2011	US	MISCAN	50-80	1410.575	0.096		14,693.49	1,509.217	0.096		15,721.009	-	-	-	-	-	-	-	-	-	-	-	-
Knudsen 2010	US	Sim-CRC	65-80	-347.712<br>-266.332	0.086<br>0.093		**CS**[b]<br>**CS**[c]	-152.895	0.092		**CS**	-	-	-	-	-	-	-	-	-	-	-	-
Lansdorp-Vogelaar 2010[a]	US	Sim-CRC	65-80	-	-	-	-	-	-	-	-	-232.984	0.078		**CS**[f]	-181.127	0.078		**CS**[f]	-	-	-	-
Vanness 2011[d]	US	Sim-CRC	50-80	130.700	0.138		947.103	252.769	0.138		1,831.661	-	-	-	-	-	-	-	-	-	-	-	-
Ladabaum 2013	US	Markov	50-80	51.587		0.079	652.180	55.105		0.081	678.630	-	-	-	-	-	-	-	-	-	-	-	-
Ladabaum 2018	US	Markov		536.252		0.079	6,779.415	800.803		0.081	9,862.101	-	-	-	-	-	-	-	-	-	-	-	-
Melnitchouk 2018	UA	Markov	50-75	-825.397		0.082	**CS**	-	-	-	-	-	-	-	-	-	-	-	-	-	-	-	-
Telford 2010	CA	Markov	50-75	1,185.647		0.106	11,185.349	-	-	-	-	-	-	-	-	-	-	-	-	-	-	-	-
Wong 2016	HK	Markov	50-70	-	-	-	-	-	-	-	-	-	-	-	-	-	-	-	-	1,479.835<br>1,474.435	0.038<br>0.035		38,527.338[g]<br>42247.433[h]

FR: France; US: United States of America; SG: Singapore; CA: Canada; HK: Hong Kong; UK: United Kingdom; AU: Australia; IT: Italy; IE: Ireland; UA: Ukraine
CS: cost saving, when a strategy was less costly and equally or more effective
D: dominant, when a strategy was equally or more costly and less effective
LYG: life years gained
QALY: quality-adjusted life years
ICER: incremental cost-effectiveness ratio
FOBT: guaiac-based fecal occult blood test
FIT: fecal immunochemical tests

[a] Identifical model and input data to Knudsen 2010, therefore only addtional results to Knudsen 2010 reported
[b] Hemoccult II Test
[c] Hemoccult SENSA
[d] Sigmoidoscopy without biopsy
[e] biennial FOBT/FIT
[f] triennial FOBT/FIT
[g] Sigmoidoscopy only for female patients at 50- and 55-year old followed by colonoscopy at 60- and 70-year old; male patients received colonoscopy at 50-, 60-, and 70-year old
[h] Sigmoidoscopy only for female patients at 50-, 55-, 60, and 65-year old followed by colonoscopy at 70-year old; male patients received colonoscopy at 50-, 60-, and 70-year old

Table A5 Incremental cost, life years gained, quality-adjusted life years and incremental cost-effectiveness ratios of 5-yearly sigmoidoscopy combined with biennial/triennial stool tests compared with sigmoidoscopy or colonoscopy (in 2019 $)

				**5-yearly sigmoidoscopy + biennial/triennial FOBT**								**5-yearly sigmoidoscopy + biennial/triennial FIT**							
			Screening	vs. 10-yearly colonoscopy				vs. 5-yearly sigmoidoscopy				vs. 10-yearly colonoscopy				vs. 5-yearly sigmoidoscopy			
Author, Year	Country	Model Name	Period	ΔCost	LYG	ΔQALY	ICER	ΔCost	LYG	ΔQALY	ICER	ΔCost	LYG	ΔQALY	ICER	ΔCost	LYG	ΔQALY	ICER
Barzi 2017	US	Markov	50-75	287.998	-0.008		**D**[e]	-18.337	-0.002		9,168.490[e]	426.065	-0.010		**D**[e]	119.730	-0.004		**D**[e]
Lansdorp-Vogelaar 2010[a]	US	MISCAN	65-80	-133.097	-0.003		42,934.564[f]	-30.880	0.009		**CS**[f]	32.76875	-0.0033		**D**[f]	134.9861	0.0086		15,696.062[f]
Lansdorp-Vogelaar 2010[a]	US	Sim-CRC	65-80	-114.861	-0.008		15,113.260[f]	-101.667	0.019		**CS**[f]	-63.004	-0.008		7,875.455[f]	-49.810	0.019		**CS**[f]

US: United States of America
CS: cost saving, when a strategy was less costly and equally or more effective
D: dominant, when a strategy was equally or more costly and less effective
LYG: life years gained
QALY: quality-adjusted life years
ICER: incremental cost-effectiveness ratio
FOBT: guaiac-based fecal occult blood test
FIT: fecal immunochemical tests

[a] Identifical model and input data to Knudsen 2010, therefore only addtional results to Knudsen 2010 reported
[e] biennial FOBT/FIT
[f] triennial FOBT/FIT

Table A6 Incremental cost, life years gained, quality-adjusted life years and incremental cost-effectiveness ratios of sigmoidoscopy in other combinations compared with no screening (in 2019 $)

Author, Year	Country	Model Name	Screening Period	**10-yearly sigmoidoscopy + annual FOBT vs. No Screening**				**10-yearly sigmoidoscopy + annual FIT vs. No Screening**				**Sigmoidoscopy once + FOBT vs. no screening**				**Sigmoidoscopy once + FIT vs. no screening**				**Sigmoidoscopy once + 10-yearly colonoscopy vs. no screening**			
				∆Cost	LYG	∆QALY	ICER	∆Cost	LYG	∆QALY	ICER	∆Cost	LYG	∆QALY	ICER	∆Cost	LYG	∆QALY	ICER	∆Cost	LYG	∆QALY	ICER
Dinh 2013	US	Archimedes	50-75	-	-	-	-	-1,486.66		0.112	**CS**	-	-	-	-	-	-	-	-	-	-	-	-
Naber 2019	US	CRC-SPIN	65-75	-1,500.86	0.099		**CS**	-1,404.91	0.099		**CS**	-	-	-	**-**	-	-	-	**-**	-	-	-	**-**
Naber 2019	US	MISCAN	65-75	-228.415	0.0987		**CS**	-191.910	0.099		**CS**	-	-	-	-	-	-	-	-	-	-	-	-
Naber 2019	US	Sim-CRC	65-75	-838.564	0.099		**CS**	-798.931	0.099		**CS**	-	-	-	**-**	-	-	-	**-**	-	-	-	**-**
Ladabaum 2019	US	Markov	45-75	-	-	-	-	-	-	-	-	-	-	-	**-**	-1,211.57		0.141	**CS**[b]	-100.794		0.140	**CS**[e]
Lew 2018	AU	Policy1-Bowel	50-74	-	-	-	-	-	-	-	-	-	-	-	-	-140.088	0.0001		**CS**[c]	-	-	-	-
Whyte 2012	UK	Markov	55-74	-	-	-	-	-	-	-	-	10.8439	0.033	0.028	328.603/LYG 387.283/QALY[a]	-9.17562	0.06	0.0498	**CS**[d]	-	-	-	-

US: United States of America; UK: United Kingdom; AU: Australia
CS: cost saving, when a strategy was less costly and equally or more effective
D: dominant, when a strategy was equally or more costly and less effective
LYG: life years gained
QALY: quality-adjusted life years
ICER: incremental cost-effectiveness ratio
FOBT: guaiac-based fecal occult blood test
FIT: fecal immunochemical tests

[a] Sigmoidoscopy once at 55 + biennia FOBT (66-74)
[b] Sigmoidoscopy once at 45 + annual FIT
[c] Sigmoidoscopy once at 55 + biennial FIT
[d] Sigmoidoscopy once at 55 + biennial FIT (56-74)
[e] Sigmoidoscopy once at 45 + colonoscopy every 10 years
